# Atomistic-to-Macroscopic Mechanical Modeling of (HfNbTaZr)B_2_ High-Entropy Ceramics via a Developed Interatomic Potential

**DOI:** 10.3390/ma19194209

**Published:** 2026-10-02

**Authors:** Yihan Wu, Yuan-Xin Dai, Xingjie Chen, Pengfei Yu

**Affiliations:** 1 School of Mechanical Engineering and Automation, Fuzhou University, Fuzhou 350108, China; 2 Key Laboratory of High-Performance Polymer Materials and Technology of MOE, Department of Polymer Science and Engineering, School of Chemistry and Chemical Engineering, Nanjing University, Nanjing 210023, China; yuanxin.dai@foxmail.com; 3 State Key Laboratory for Strength and Vibration of Mechanical Structures, School of Aerospace Engineering, Xi’an Jiaotong University, Xi’an 710049, China; chenxj@stu.xjtu.edu.cn

**Keywords:** high-entropy diborides, analytical bond-order potential, chemical short-range order, nanoindentation, multiscale modeling

## Abstract

Atomistic investigations of high-entropy diborides (HEBs) are currently hindered by the lack of reliable interatomic potentials. Here, we develop an analytical bond-order potential (ABOP) for the Hf/Nb/Zr/Ta/B system, parameterized via an intelligent global optimization algorithm and trained against extensive ab initio data. The developed potential accurately captures the structural, elastic, and mechanical properties of various materials, including metals, intermetallics, alloys and diborides. Additionally, the chemical short-range order (CSRO) in dynamic simulations under finite temperatures is correctly predicted. Molecular dynamics (MD) simulations reveal that HEB nanopillars exhibit brittle failure driven by shear banding, while CSRO shows limited influence on the mechanical response. Simulated nanoindentation gives modulus (435 GPa) and hardness (27.7 GPa) consistent with theoretical predictions and experiments. Furthermore, we establish a multiscale framework by extracting an atomic traction–separation law and rescaling it based on fracture energy conservation (*G*_c_ = 21.01 J/m^2^) for finite element cohesive zone simulations. This approach captures the brittle intergranular failure of polycrystalline HEBs, effectively bridging atomic-level mechanisms with macroscopic property predictions.

## 1. Introduction

In recent years, the concept of entropy stabilization has guided the development of various multi-component materials. By combining several elements in equal or similar atomic ratios, the increased configurational entropy helps to form stable solid solutions [1,2,3]. The resulting features (e.g., lattice distortion, sluggish diffusion and the cocktail effect) generally improve the physical and mechanical performance of the material [4]. Following this principle, high-entropy alloys (HEAs) have been first designed and investigated [2,3]. Compared to traditional alloys, HEAs commonly display better strength–ductility synergy [5], radiation resistance [6], and good cryogenic [5] and catalytic properties [7]. However, the use of HEAs in high-temperature environments is sometimes limited by high-temperature softening and oxidation issues [8,9].

To meet the material requirements for more extreme environments (e.g., higher temperature), researchers applied the entropy-stabilization approach to nonmetallic materials, developing high-entropy ceramics (HECs) [10,11]. These materials are generally synthesized from mixtures of basic oxides [11], nitrides [10], carbides [4,10], or diborides [12]. In HECs, the random distribution of metal cations [6,13] and the shielding effect of the anion network [1,4,11,14] contribute to good phase stability. Particularly, the high-entropy diborides (i.e., HEBs with the AlB_2_ lattice structure) are found to maintain their hardness, strength, oxidation resistance and a certain degree of ductility at ultrahigh temperatures. Because of these thermal and mechanical features, HEBs provide a practical alternative to conventional ultrahigh-temperature ceramics and refractory alloys, showing potential for use in demanding fields like aerospace and nuclear engineering [1,15,16].

Research on HEBs is currently at an early stage. Because these materials are relatively difficult to synthesize, many studies have focused on improving preparation methods to achieve dense and uniform structures [17,18,19]. While basic material properties (e.g., thermal conductivity [20], elastic constants [16,21], and oxidation resistance [22,23,24]) have been tested and reported, the relationship between their nanoscale structures and macroscopic properties has not been fully investigated. Detailed atomic-level factors, including chemical short-range order (CSRO), elemental diffusion, and defect behavior, are still not well understood. Without such nanoscale information, predicting material performance can be difficult. Current predictions often rely on simple empirical methods [25,26,27], such as the rule of mixing, entropy forming ability, or valence electron concentration. However, the predicted values can differ from experimental results, and materials with similar empirical parameters may show different actual properties [28]. This inconsistency makes it harder to design HEBs for specific applications.

Molecular dynamics (MD) simulations provide a useful way to study atomic-level mechanisms. To perform reliable MD simulations, accurate interatomic potentials are needed to describe the interactions among different elements. Recently, machine learning (ML) models have been used to develop these potentials [29,30,31,32]. While ML potentials usually match their training data well, their data-driven nature can limit their accuracy when applied to situations outside their training set. As a result, they may be less capable of simulating dynamic or non-equilibrium processes, such as large deformation, crack growth, dislocation movement, or radiation damage. By comparison, classical empirical or semi-empirical potentials are based on physical models [8,9,14,33], which generally helps them perform more stably in new or extreme conditions outside the training domain. The main difficulty in developing classical potentials for HEBs is the complex bonding environment, which involves a mix of metallic, ionic, and covalent bonds [14,15]. Describing these mixed interactions requires mathematical formulas that account for bond angles and bond orders. These formulas contain a large number of internal parameters, making the fitting process too complicated for conventional optimization methods [9,14,33].

To enable MD simulations for the HEB materials, this study develops an analytical bond-order potential (ABOP) for the Hf/Nb/Zr/Ta/B exemplified system. To handle the complex parameter fitting process, we use a modified distributed breeder genetic algorithm (DBGA) based on ab initio reference data. The remainder of this paper is organized as follows. Section 2 introduces the ABOP model and explains the parameter optimization procedure. Section 3 evaluates the accuracy of the developed potential by comparing it with ab initio reference data. In Section 4, we apply the potential to several typical dynamic simulations to test its practical performance. Overall, this work provides a basic tool for studying the nanoscale mechanical behaviors and the associated atomic mechanisms of high-entropy ceramics.

## 2. Computational Details

### 2.1. The Analytical Bond-Order Potential (ABOP)

The fidelity of atomistic simulations depends on an interatomic potential that accurately reflects the underlying bonding nature of the material. Given the pronounced directional covalent bonding in high-entropy ceramics such as (HfNbTaZr)B_2_, an angular-dependent formulation that captures local bond-order effects is essential. Here, we implement the analytical bond-order potential (ABOP) framework [14,34], defining the total potential energy as follows:

(1) $$V=\sum_{i,j}V_{\mathrm{ij}}=\frac{1}{2}\sum_{i,j}f_{c}\left(r_{\mathrm{ij}}\right)\left[V_{R}\left(r_{\mathrm{ij}}\right)-b_{\mathrm{ij}}V_{A}\left(r_{\mathrm{ij}}\right)\right],$$

where *r_ij_* denotes the interatomic distance, *f_c_*(*r_ij_*) is a smooth cutoff function, *V_R_*(*r_ij_*) and *V_A_*(*r_ij_*) represent the pairwise repulsive and attractive contributions, respectively. The many-body environment is implicitly encoded in the bond-order term *b_ij_*, which incorporates local coordination numbers and bond-angle dependencies. This mathematical architecture enables ABOP to model complex, hybridized metallic–covalent bonding within a unified framework.

While ABOP has been successfully parameterized for various intermetallic, ionic, and covalent systems [34,35], abrupt cutoff truncations frequently induce unphysical artificial ductility and overestimate the theoretical strength during tensile or fracture simulations [14]. To resolve this artifact, the cutoff parameters in *f*_c_ are dynamically refined during parameter optimization. Additionally, to accurately capture high-energy collision cascades and close-contact interactions, the short-range repulsive domain is smoothly connected to the Ziegler–Biersack–Littmark (ZBL) potential following the strategy reported previously [14].

### 2.2. Training Data Generation

To parameterize the ABOP, a comprehensive reference dataset containing elemental metals, intermetallic compounds, and diborides is constructed via ab initio calculations. The representative configurations and corresponding target properties are summarized in Table 1. Notably, atomic virial stress tensors are incorporated as an independent fitting objective, which is a strategy demonstrated to substantially improve the robustness and mechanical transferability of interatomic force fields [36]. Benefiting from the physically motivated functional form, the ABOP requires a significantly more compact dataset than typical ML potentials [9,14].

All density functional theory (DFT) calculations are performed using the Vienna Ab Initio Simulation Package (VASP) [37] within the projector augmented-wave (PAW) framework. Electronic exchange-correlation effects are treated using the Perdew–Burke–Ernzerhof (PBE) generalized gradient approximation (GGA) [38]. The plane-wave cutoff energy is set to 520 eV, and ionic relaxations are converged until the Hellmann–Feynman forces are below 10^−4^ eV/Å. The Brillouin zone is sampled using Γ-centered Monkhorst–Pack k-point grids generated via VASPKIT with a k-mesh resolution of 0.02 × 2π Å^−1^ [39]. Initial structural prototypes are retrieved from the Open Quantum Materials Database (OQMD) [40].

### 2.3. Global Optimization

With the ab initio database established, parameterizing the quinary Hf/Nb/Zr/Ta/B ABOP requires solving a 254-dimensional optimization problem. The optimal parameters are determined by minimizing a normalized objective loss function (i.e., ∆), formulated as follows:

(2) $$\begin{array}{l} \Delta =\sum_{i}\frac{\omega_{i}{\left(E_{\mathrm{ABOP},i}-E_{\mathrm{ab}\;\mathrm{initio},i}\right)}^{2}}{E_{\mathrm{ab}\;\mathrm{initio},i}^{2}}+\sum_{j}\sum_{\alpha }\frac{\omega_{j}{\left(S_{\mathrm{ABOP},j,\alpha }-S_{\mathrm{ab}\;\mathrm{initio},j,\alpha }\right)}^{2}}{S_{\mathrm{ab}\;\mathrm{initio},j,\alpha }^{2}}\\ \;\;\;+\sum_{k}\sum_{\beta }\frac{\omega_{k}{\left(M_{\mathrm{ABOP},k,\beta }-M_{\mathrm{ab}\;\mathrm{initio},k,\beta }\right)}^{2}}{M_{\mathrm{ab}\;\mathrm{initio},k,\beta }^{2}} \end{array},$$

where *E*, *S*, and *M* denote energetic, structural (e.g., lattice vectors, atomic coordinates), and mechanical quantities (e.g., elastic constants, virial stress components), with indices (*i*, *j* and *k*) covering the respective sizes of dataset. The directional indices *α* = 1~3 and Voigt stress components *β* = 1~6 resolve spatial and tensorial dimensions. Although weight factors *ω* critically govern both convergence rate and predictive power [9,14,33], their assignment is often challenging due to disparate physical dimensions and orders of magnitudes. By employing a relative error normalization in Equation (2), magnitude disparities across different properties are reduced. Therefore, the default weights are set to unity (*ω* = 1), except for cohesive energies, which receive an increased weighting of *ω* = 10 to emphasize phase stability in dynamic simulations.

**Figure 1:**
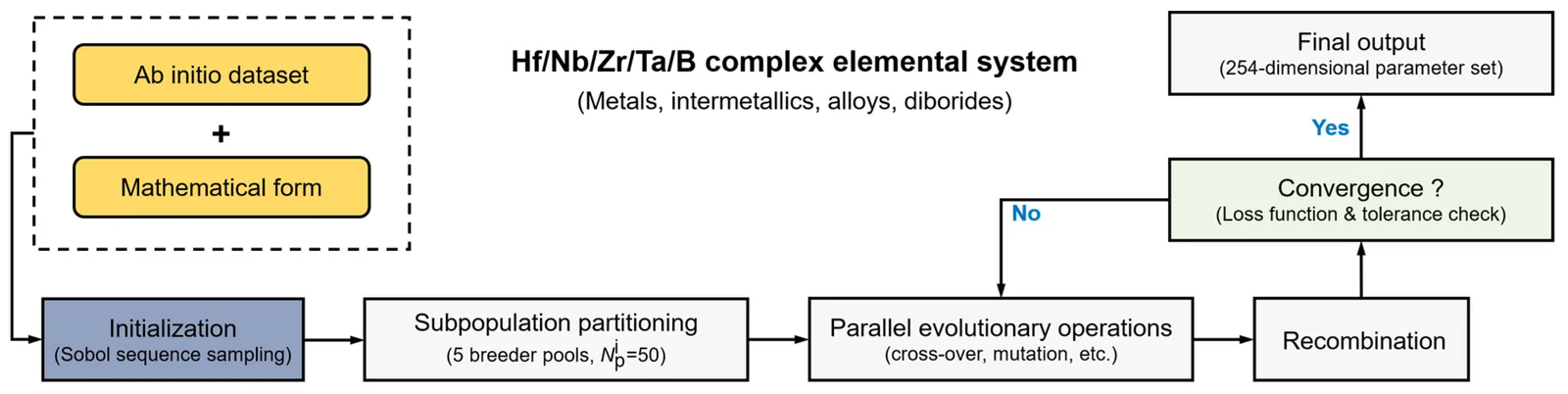
The workflow of ABOP parameterization.

Searching such an ultrahigh-dimensional, non-convex parameter landscape makes gradient-based approaches (e.g., conjugate-gradient or steepest-descent methods) susceptible to convergence to spurious local minima [38,41]. Therefore, we employ a derivative-free global optimizer based on a modified distributed breeder genetic algorithm (DBGA) [9,14,33], which accelerates convergence by emulating artificial selection principles. As depicted in Figure 1, the workflow initializes a population of *N*_P_ = 250 candidate parameter sets, sampled via a quasi-random Sobol sequence [42]. This sampling method guarantees high parameter space coverage and initial genetic diversity. Thereafter, the generated population is partitioned into five parallel subpopulations (so-called breeder pools) for localized evolutionary operations, including tournament selection, extended intermediate recombination, and a two-stage hybrid (i.e., uniform and breeder) mutation [9,14]. This dual-mutation strategy balances broad re-initialization with fine local perturbation, thus preventing premature stagnation, particularly in late-stage runs. Finally, the independent breeder pools are merged, and the top *N*_P_ individuals ranked by fitness (defined as 1/∆) are retained to initialize the next generation. This process iterates until the predefined convergence criteria are met. More details of the global optimization procedure can be found in Refs. [9,14,33].

The interatomic potential developed in this work is available in the authors’ GitHub repository at https://github.com/wuyihan1995/Analytical-bond-order-potential-ABOP-High-entropy-ceramics. (accessed on 27 September 2026).

### 2.4. Molecular Dynamics (Md) Simulations

All atomistic simulations are conducted within the LAMMPS package [43] using a 1.0 fs timestep. A Nosé–Hoover thermostat and barostat are utilized to control the temperature and pressure of the simulation system. Post-processing and visualization are carried out via OVITO software [44]. Specifically, local crystalline lattice and phase transformations are monitored using Polyhedral Template Matching (PTM) [45].

## 3. Results

### 3.1. Performance in Metallic Systems

**Figure 2:**
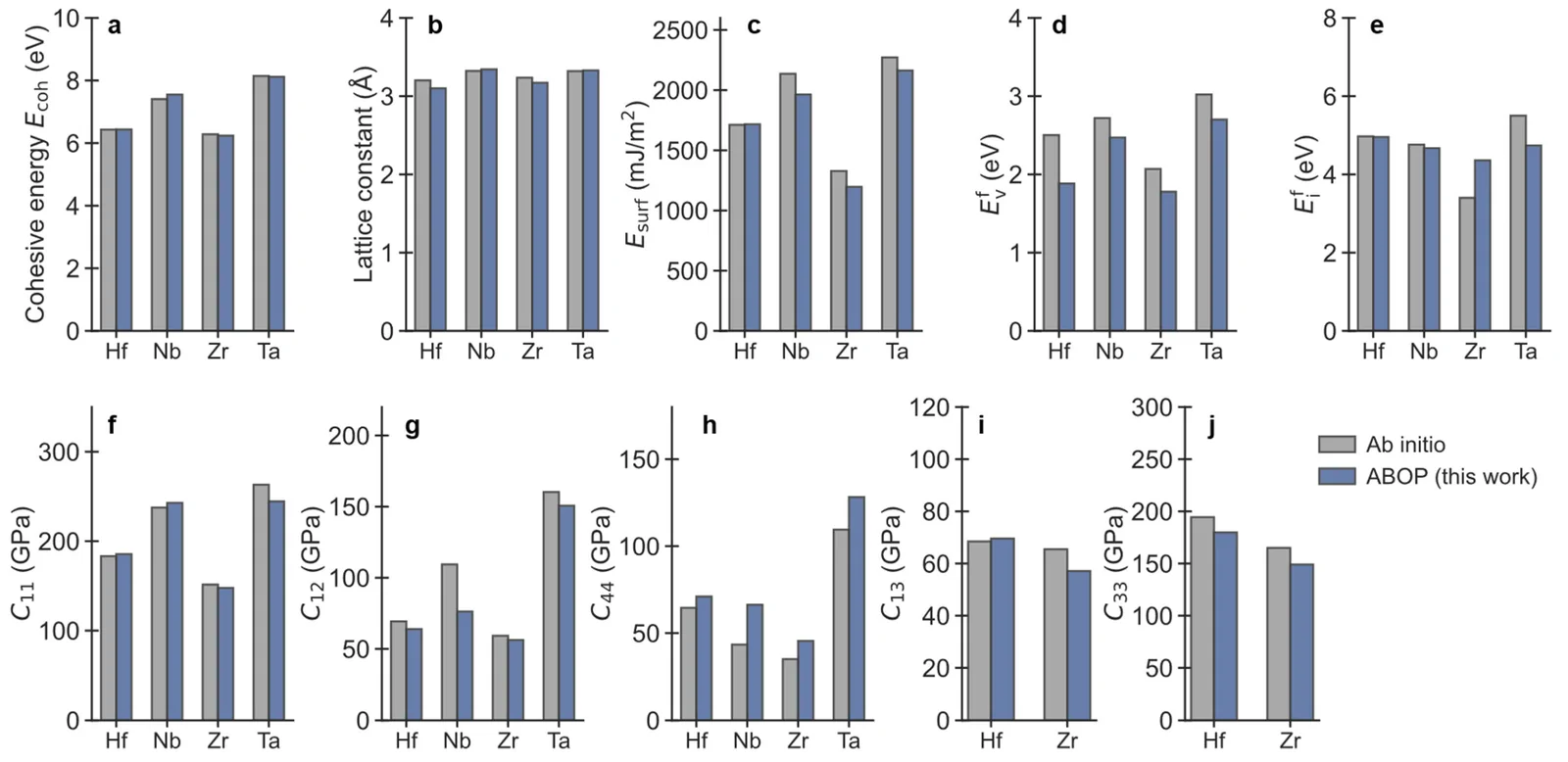
The ABOP-predicted properties of elemental metals. Ab initio data are shown for comparison. The data contain (**a**) cohesive energy *E*_coh_, (**b**) lattice constant, (**c**) surface energy *E*_surf_, (**d**) vacancy formation energy *E*^f^_v_, (**e**) interstitial formation energy *E*^f^_i_ and (**f**–**j**) independent elastic constants *C*_11_~*C*_44_.

**Figure 3:**
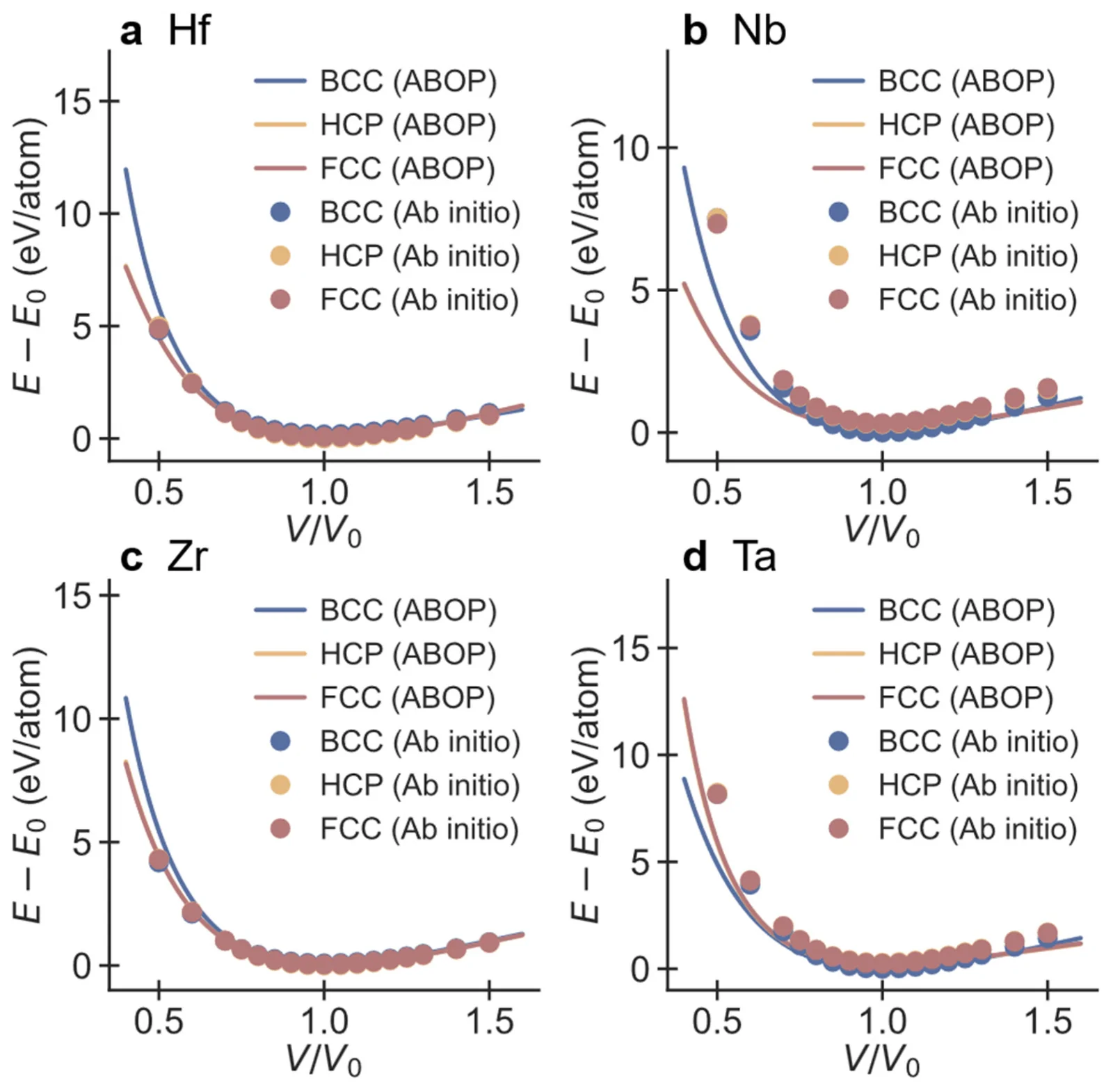
The equations of state (EOSs) of HCP, BCC and FCC polymorphs for (**a**) Hf, (**b**) Nb, (**c**) Zr and (**d**) Ta. *E*_0_ and *V*_0_ denote the per-atom energy and cell volume at the stress-free state, respectively.

The predictive performance of the developed ABOP for elemental Hf, Nb, Zr, and Ta is summarized in Figure 2 and Figure 3, compared with data from ab initio calculations. Apparently, the ABOP correctly reproduces structural, energetic and mechanical properties of the four metallic materials. Notably, the formation energies of defects (i.e., surface, vacancies and interstitials) are not included in the training process, but the developed potential still captures these properties in good agreement with ab initio results (Figure 2c–e). Such a cross-validation strategy effectively avoids overfitting, thereby elevating the transferability of the ABOP dynamic simulations. In addition, the equations of state (EOSs) predicted by the ABOP are also consistent with ab initio data. The potential accurately tracks the energy profiles across various crystalline polymorphs under wide hydrostatic strains (±50%, Figure 3), thus precluding spurious local minima near the equilibrium and preserving structural robustness during dynamic simulations [9,14,33].

**Figure 4:**
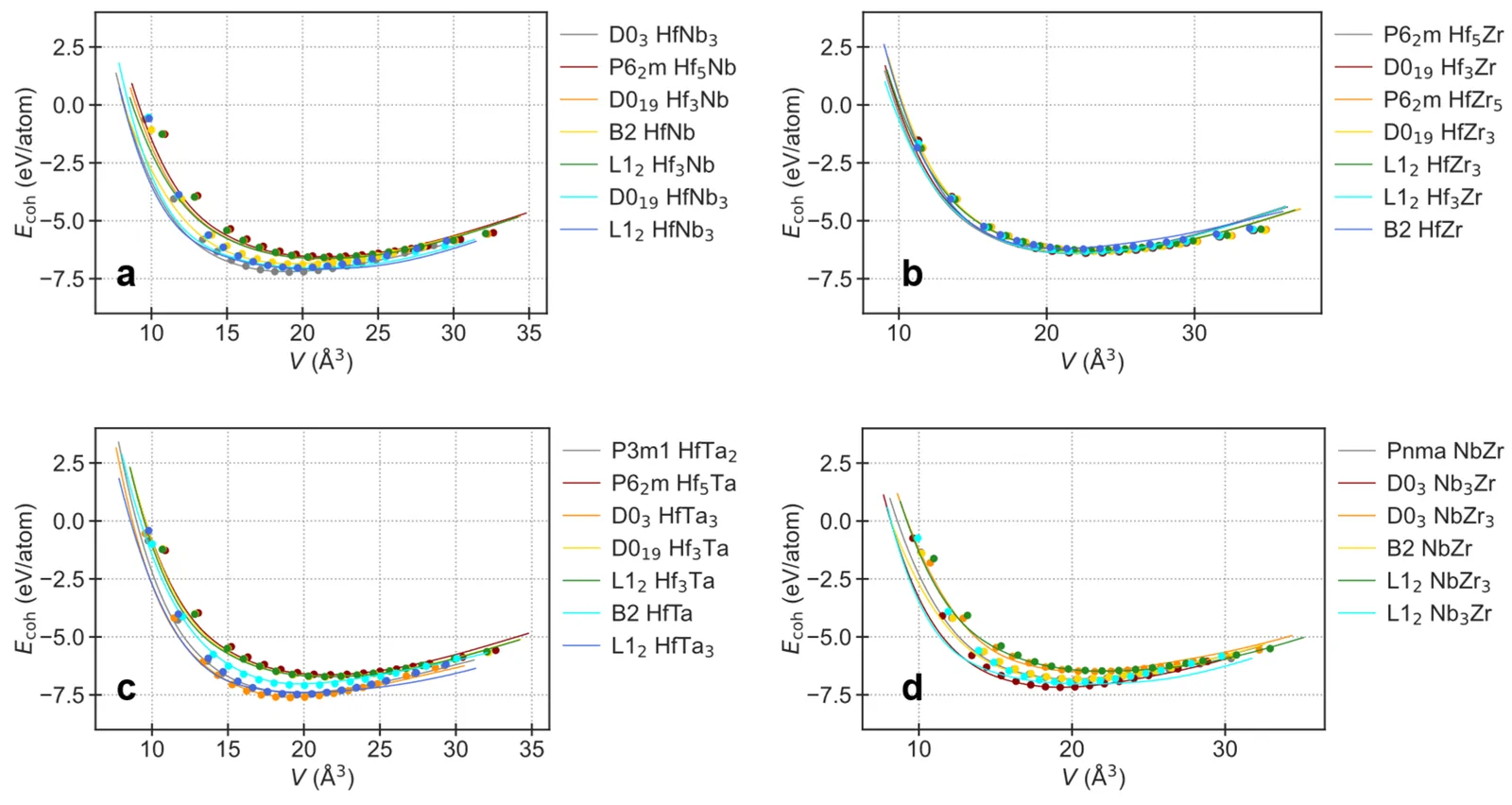

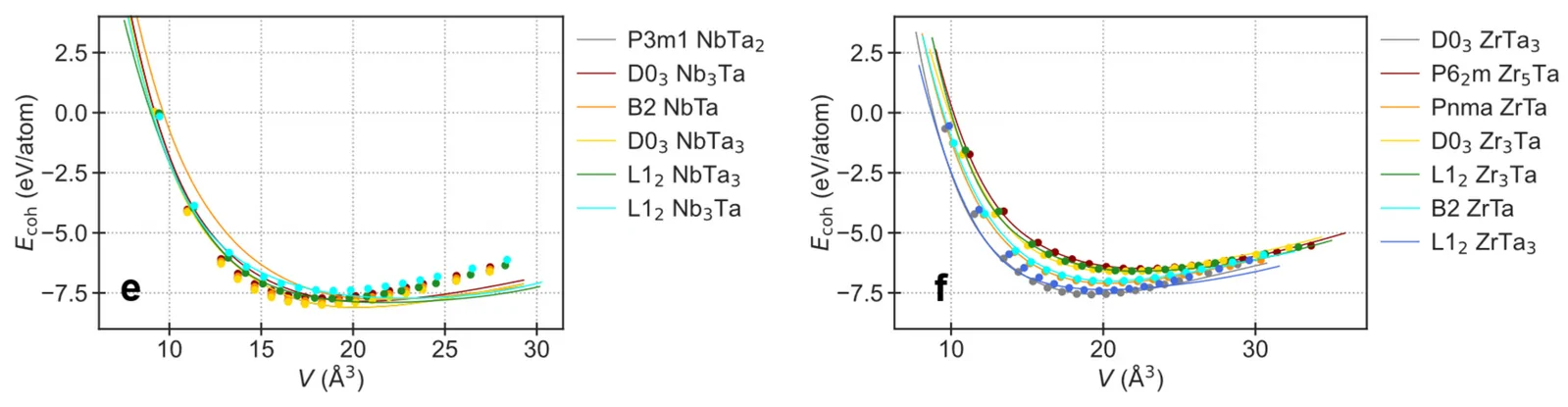
The equation of state (EOS) curves of various binary intermetallic compounds (**a**) Hf-Nb, (**b**) Hf-Zr, (**c**) Hf-Ta, (**d**) Nb-Zr, (**e**) Nb-Ta and (**f**) Zr-Ta, calculated using ABOP (lines) in comparison to ab initio results (dots).

**Figure 5:**
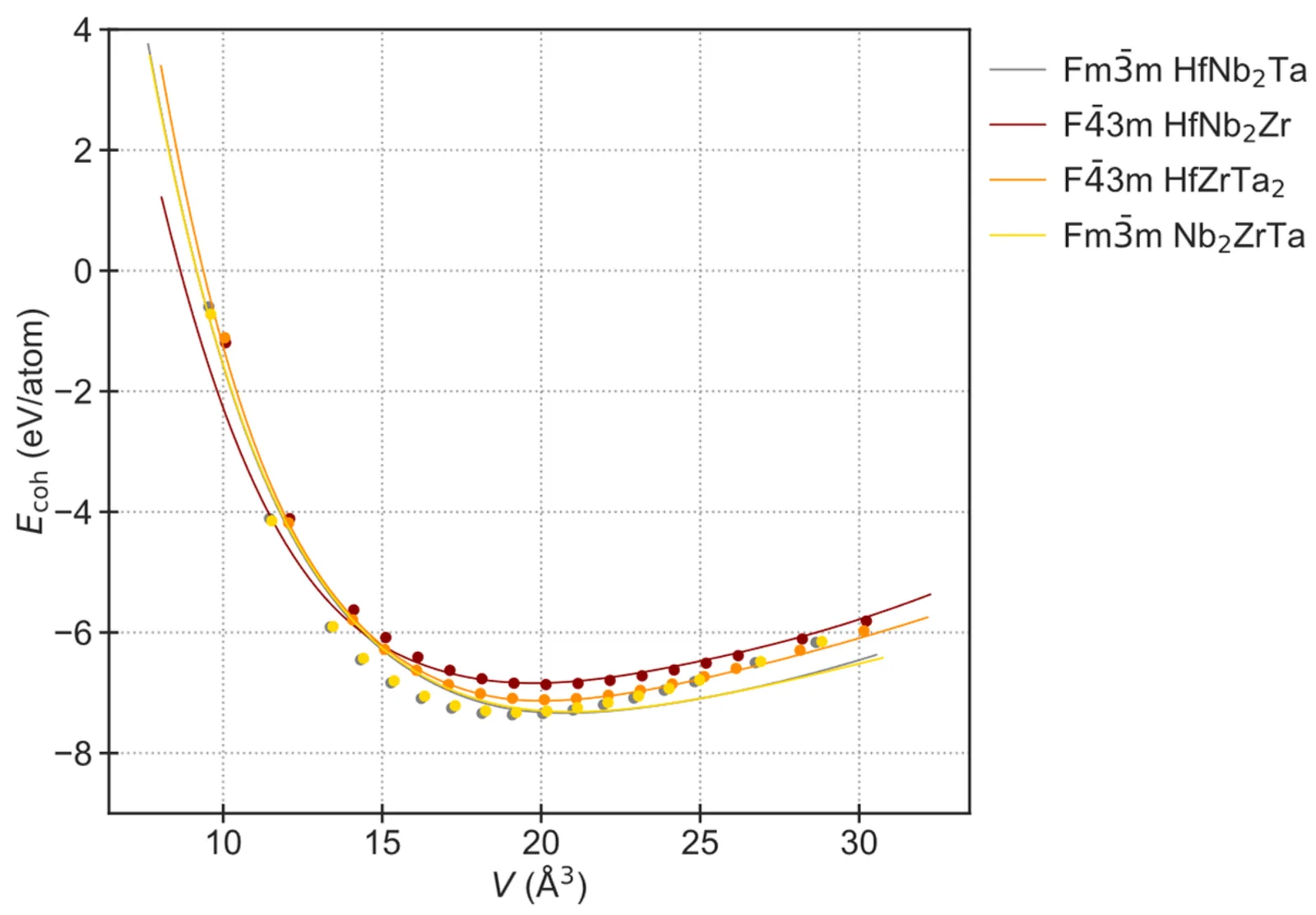
The EOS curves of some ternary intermetallic compounds predicted using ABOP (lines) and ab initio method (dots).

The EOS curves for some binary intermetallics with various structural phases (e.g., B2, L1_2_, D0_3_, D0_19_, etc.) are presented in Figure 4. The developed potential accurately describes both the ground-state energies and the energetic responses under finite volumetric dilation and compression. Furthermore, to assess the ABOP’s predictive capacity beyond the training domain, calculations are extended to ternary compounds (Figure 5). The resulting EOS profiles match the ab initio reference data with high accuracy, which indicates that this potential successfully learns the underlying many-body cross-interactions. This result supports the ability of the ABOP to describe compositional environments beyond the binary compounds included in the fitting dataset.

**Figure 6:**
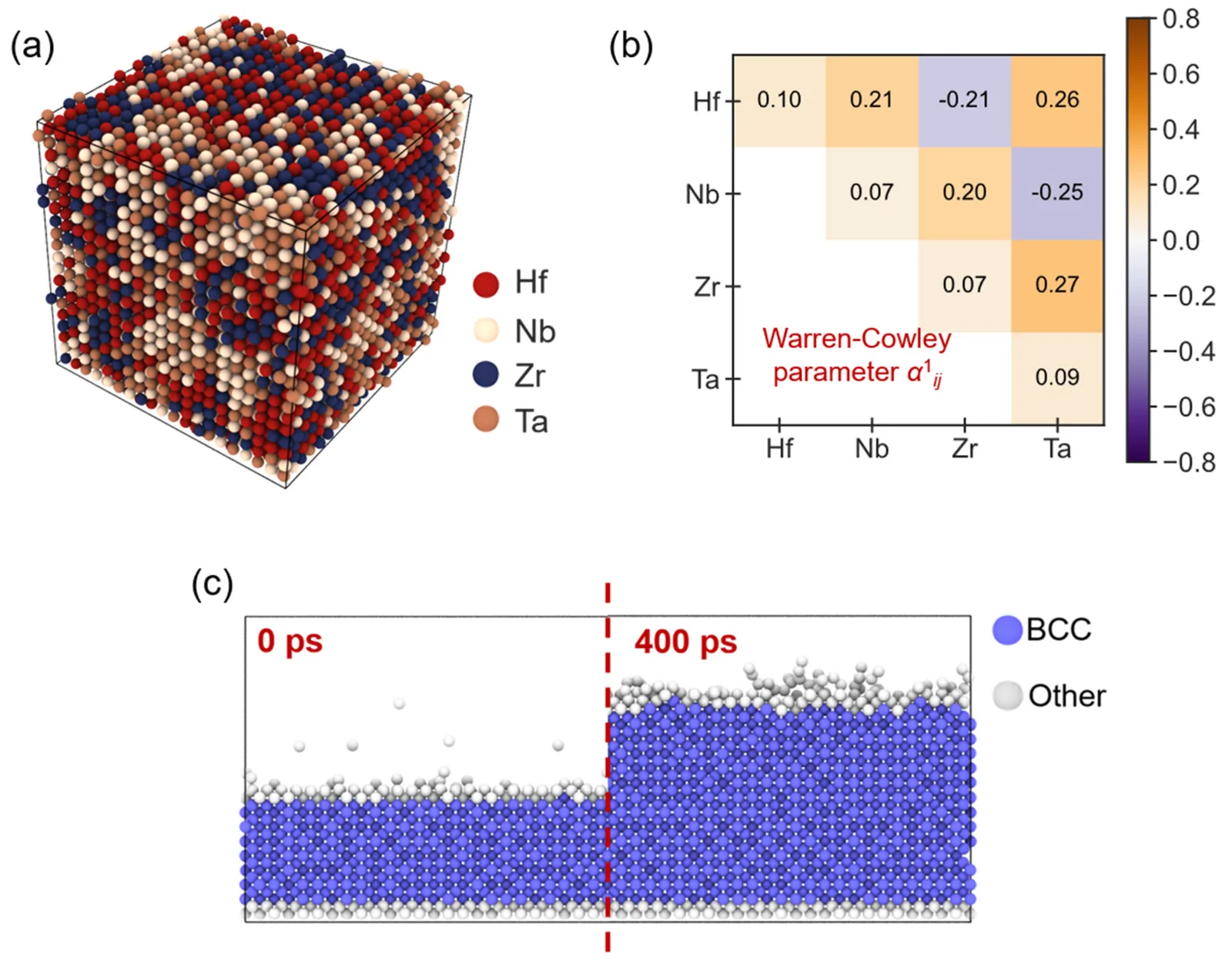
(**a**) The MC-optimized HEA configuration. (**b**) The corresponding Warren–Cowley order parameters illustrating chemical short-range order. (**c**) Simulation results of atomic deposition.

Apart from static target properties, evaluating the ability of the potential to preserve structural stability during finite-temperature dynamical simulations is essential. As reported in previous studies [46,47], complex potentials with flexible mathematical forms and high parameter counts (e.g., ML-based and bond-order formalisms) often suffer unphysical structural breakdown in dynamic simulations, even though they accurately describe static DFT reference data. To examine this dynamic stability, chemical short-range order (CSRO) in quaternary HfNbZrTa HEA is studied at room temperature. The bulk HEA maintains its BCC lattice structure throughout the Monte-Carlo relaxation at 300 K. An apparent local clustering of Hf-Zr and Nb-Ta pairs is evident from both the optimized atomic configuration (Figure 6a) and the Warren–Cowley (WC) order parameters (Figure 6b). The WC parameters can be calculated using [9,14]

(3) $$\alpha_{\mathrm{ij}}^{n}=\left(p_{\mathrm{ij}}^{n}-C_{j}\right)/\left(\delta_{\mathrm{ij}}-C_{j}\right),$$

where *n* represents the *n*-th nearest-neighbor coordination shell of species *i*, *p_ij_* is the probability of encountering a *j* atom around an *i* atom, *C_j_* is the nominal concentration and *δ_ij_* is the Kronecker delta function. Negative values of the WC parameters indicate a clustering tendency for the given pair. The clustering between Hf-Zr and Nb-Ta agrees with their respective ground-state crystal phases (i.e., HCP for Hf/Zr and BCC for Nb/Ta). Moreover, the CSRO pattern is also consistent with previous experiments [48,49]. Findings in Figure 6a,b indicate that the ABOP correctly learns interactions among metals at finite temperatures.

In addition, atomic deposition simulations are also performed at room temperature (Figure 6c). The ABOP accurately captures the vapor deposition process, wherein vaporized atoms spontaneously crystallize into the equilibrium BCC lattice on the substrate. The deposition simulation further demonstrates that the developed ABOP can maintain physically realistic structural evolution under the far-from-equilibrium condition, which is a capability critical for reliable simulations of radiation cascades and shock-induced deformation [8,33]. Overall, the results in Figure 6 further support the structural robustness of the ABOP for the metallic systems and dynamic scenarios examined here.

### 3.2. Performance in Ceramic Systems

**Figure 7:**
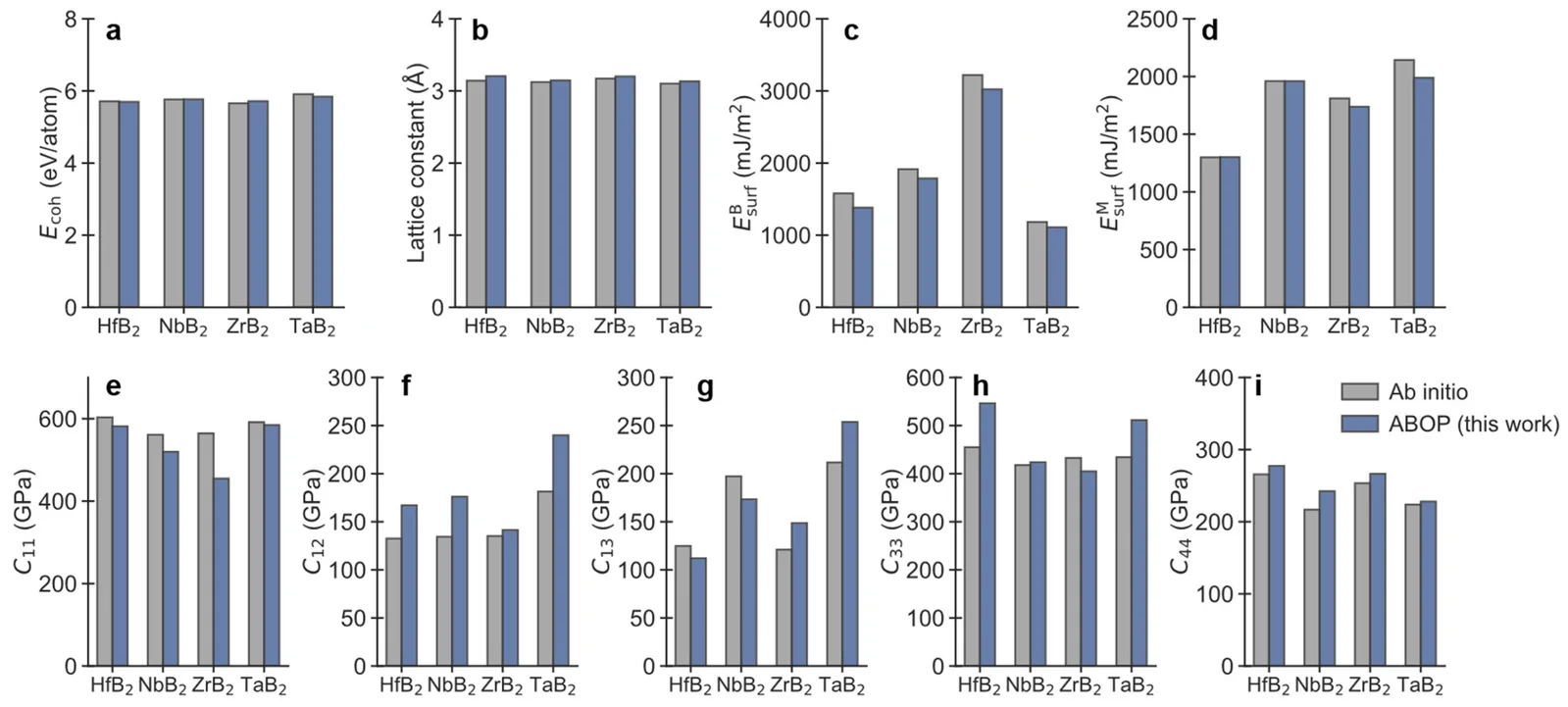
The ABOP-predicted properties of unary diborides. Ab initio data are shown for comparison. The data contain (**a**) cohesive energy *E*_coh_, (**b**) lattice constant, (**c**,**d**) boron-/metal-terminated (0001) surface energies $E_{\mathrm{surf}}^{B}/E_{\mathrm{surf}}^{M}$, and (**e**–**i**) independent elastic constants *C*_11_~*C*_44_.

The basic properties of four unary diborides—HfB_2_, NbB_2_, ZrB_2_ and TaB_2_ (hexagonal lattice, space group: P6/mmm)—calculated using ABOP and ab initio methods are displayed in Figure 7. The cohesive energies, lattice and elastic constants of these diborides are correctly reproduced by ABOP. Notably, given the particular lattice structures of the AB_2_-type diborides, they exhibit two different (0001) surfaces terminated by metals and boron atoms, respectively. Thus, a reliable potential must correctly differentiate these two surfaces and give proper predictions on the corresponding surface formation energies. This is highly relevant to the reliability of simulations involving surfaces and interfaces, such as nanopillar compression, nanoindentation, grain boundaries (GBs) and surface ion implantations. Figure 7c,d demonstrate that the ABOP can predict the metal- and boron-terminated (0001) surface energies in good agreement with ab initio data.

**Figure 8:**
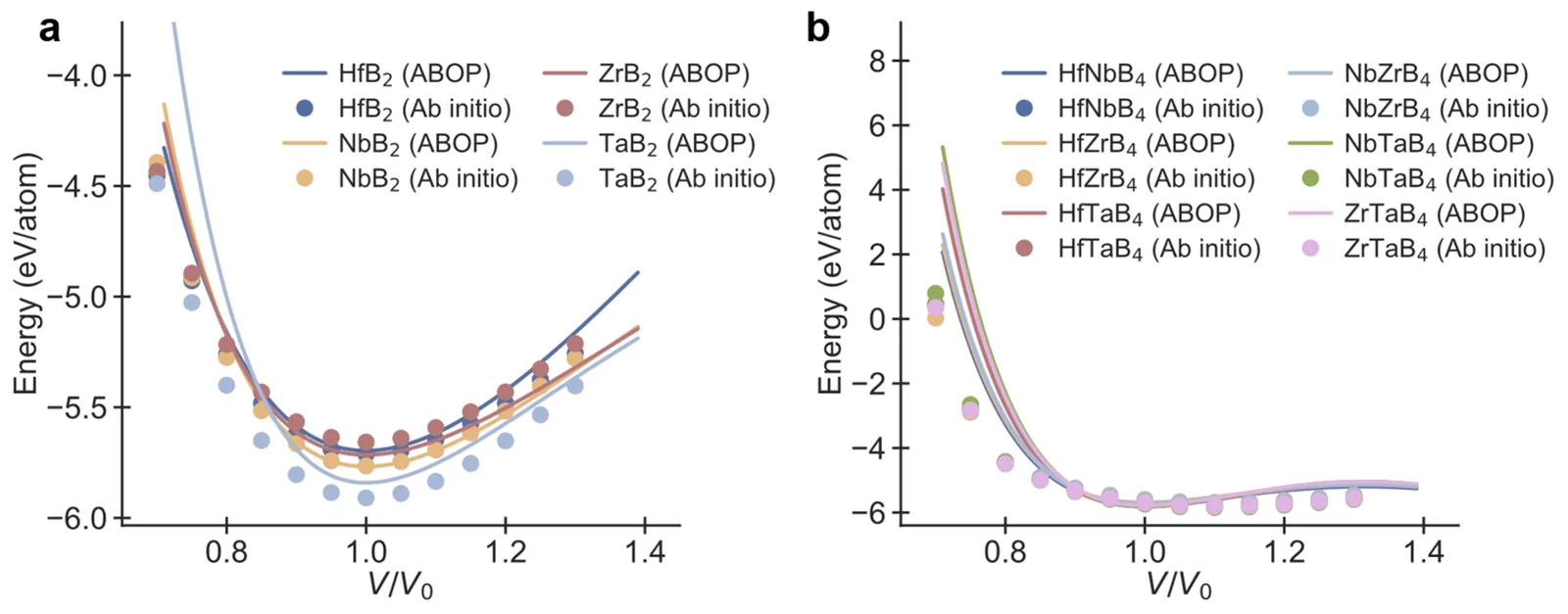
The EOS data of (**a**) unary diborides and (**b**) binary diborides calculated using ABOP (lines) and ab initio method (dots).

In addition, the EOS curves of unary diborides calculated using ABOP in comparison with ab initio data are shown in Figure 8a. The MD results are consistent with ab initio training data within a wide range of hydrostatic strain. Moreover, the EOS curves of six binary diborides are displayed in Figure 8b, which are not included in the training process. The ABOP predictions are consistent with ab initio data. This cross-validation result suggests that the developed potential is able to capture underlying physics about elemental interactions within ceramic phases.

**Figure 9:**
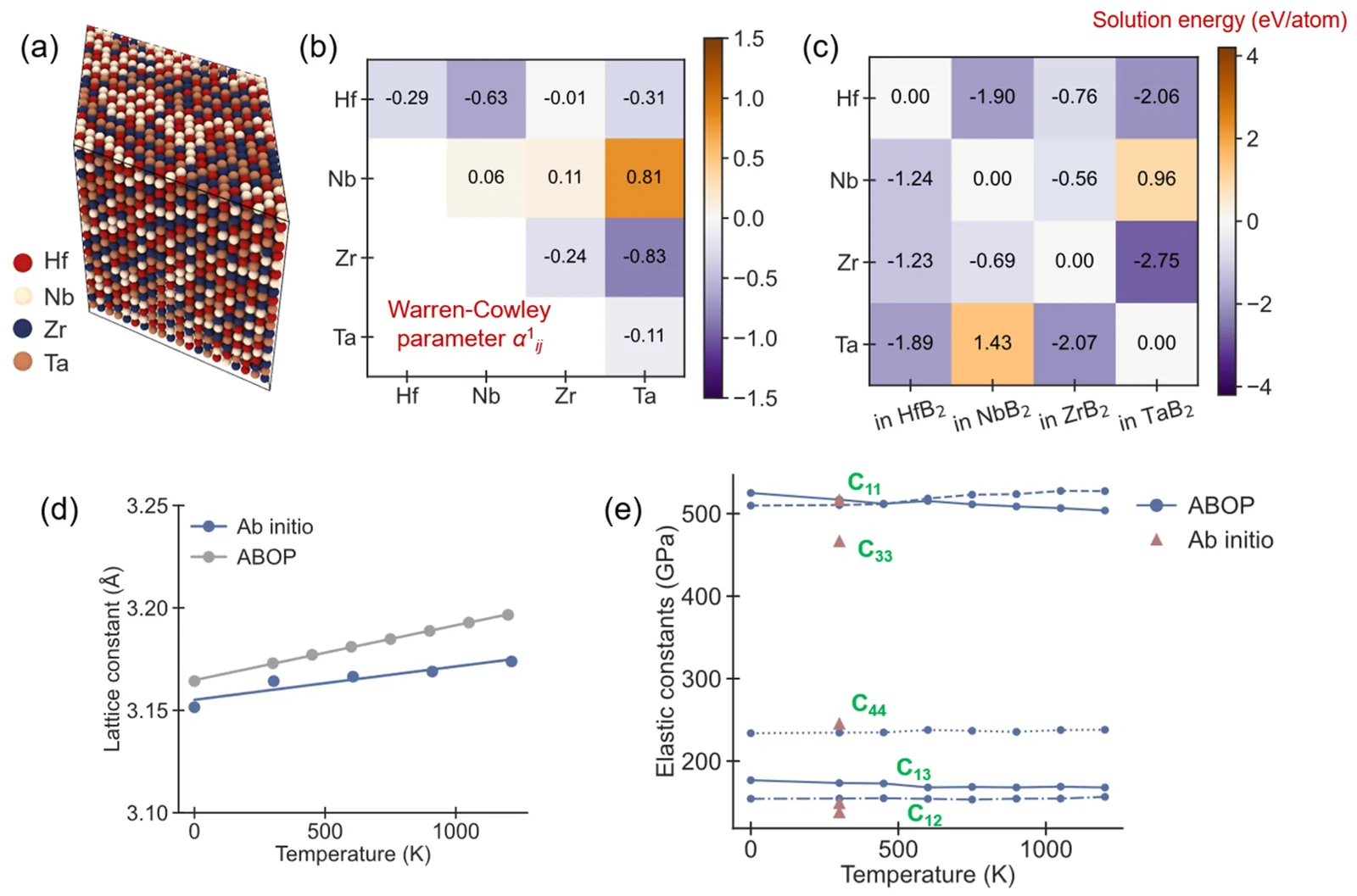
Independent validation and finite-temperature characterization of the (HfNbZrTa)B_2_ HEB, which is not included in the training data. (**a**) Distribution of metallic elements in HEB after MC optimization (boron atoms are removed); (**b**) the corresponding Warren–Cowley CSRO parameters; (**c**) the solution energies (eV/atom) of metals into different diborides. (**d**,**e**) The temperature-dependence of lattice and elastic constants of HEB; ab initio data (triangles) are shown for comparison.

According to most previous experimental investigations [1,4,11], the HEB usually exhibits outstanding stability and microscale homogeneity due to the boron anion sublattice, which screens metal cations from one another and effectively promotes configurational disorder. However, owing to the differences in atomic radii and electronegativities among the constituent transition metals, local CSRO should inherently develop at the atomistic level. Figure 9a,b display the ABOP-predicted CSRO within the HEB, which suggest Hf-Nb and Zr-Ta pairing. Such a clustering tendency among cations, influenced by the interlocked anion sublattice, is different from that within the HfNbZrTa HEA (Figure 6). It should be noted that the Warren–Cowley parameters here characterize chemical correlations within the cation sublattice; the effect of such cation rearrangement on the full local coordination environment is further examined in Section 4.1 and Figure S2 of the Supplementary Material.

To verify the formation of a cation CSRO, we evaluated the solution energies corresponding to various cation substitutions in the four unary diborides. The energetic profiles in Figure 9c reasonably explain the results in Figure 9b. Nb, Zr and Ta show the lowest solution energy in HfB_2_, TaB_2_ and ZrB_2_. Hf shows negative solution energies in NbB_2_, ZrB_2_ and TaB_2_, which is consistent with the negative WC parameters of Hf-Nb, Hf-Zr and Hf-Ta. Because CSRO is known to exert a major influence on macroscopic mechanical performance in complex concentrated systems [29,46,50], the developed ABOP provides a powerful computational tool to uncover these fine atomistic features and clarify their influence on mechanical responses.

In addition, beyond the unary and binary diborides, the full five-element (HfNbZrTa)B_2_ high-entropy diboride is directly examined as an independent validation system. This material is not included in the ABOP training dataset. Figure 9d shows that the ABOP-predicted lattice constant *a* = 3.17 Å at 300 K (*c* = 3.42 Å), corresponding to a density of 8.77 g/cm^3^, reasonably consistent with experimentally measured values [12,51]. The variation in lattice constant with temperature described by ABOP is also consistent with ab initio results. Meanwhile, the ABOP-predicted temperature-dependence of elastic constants of HEB is shown in Figure 9e. *C*_11_, *C*_12_ and *C*_13_ slightly decrease with increasing temperature, while *C*_33_ and *C*_44_ are almost invariant as temperature rises. The room-temperature elastic constants predicted by ABOP and the ab initio method are in reasonable agreement. Furthermore, the elastic constants follow the criteria of mechanical stability: *C*_12_ > 0, *C*_44_ > 0, *C*_11_ > *C*_12_, and (*C*_11_ + *C*_12_)*C*_33_ − 2*C*_13_^2^ > 0. Overall, the results in Figure 9 validate the ABOP’s applicability in dynamic simulations of complex ceramic systems at finite temperatures.

To further evaluate the ABOP outside its fitting configurations, additional independent DFT tests are performed for the quinary HEB, which is not included in the fitting database. Vacancy and surface energetics are calculated using ABOP compared with ab initio data. As listed in Table 2, the ABOP predicts an average metallic vacancy formation energy of 4.23 eV, close to 4.36 eV from ab initio calculations; the ABOP-predicted boron vacancy formation energy is 0.86 eV, compared with 0.80 eV from ab initio calculations. In addition, the ABOP gives a metal-terminated (0001) surface energy of 1.84 J/m^2^, close to the DFT-based reference of 1.80 J/m^2^. Meanwhile, the boron-terminated surface energy is 1.61 J/m^2^, compared with the 1.97 J/m^2^ DFT value. The ABOP (0001) cleavage work is 3.45 J/m^2^, compared with the 3.78 J/m^2^ DFT value. These additional tests further validate the applicability of developed ABOP in the HEB system. Moreover, additional tests are performed for ideal shear deformation and GB separation of the quinary HEB. These tests further assess the transferability of the ABOP under highly non-equilibrium mechanical loading conditions. The corresponding results, including maximum shear stresses and maximum cohesive tractions, support the applicability of the developed ABOP to nonlinear deformation and GB fracture. Details are provided in Section S5 of the Supplementary Material. Overall, these results provide independent validation of the ABOP for defect energetics, surface/interface bonding, nonlinear shear deformation, and interfacial decohesion in the target HEB.

## 4. Discussions

As presented in the previous section, the developed ABOP is able to predict the properties of elements, intermetallic compounds and diborides in the Hf/Nb/Zr/Ta/B system. Additionally, it is also proven capable of maintaining structural stability in simulations under finite temperatures. The robustness and reliability of the ABOP have been demonstrated. In this section, we further examine the performance of the ABOP in several representative mechanical simulation scenarios relevant to the target HEB.

### 4.1. Compressive Response of Nanopillars

**Figure 10:**
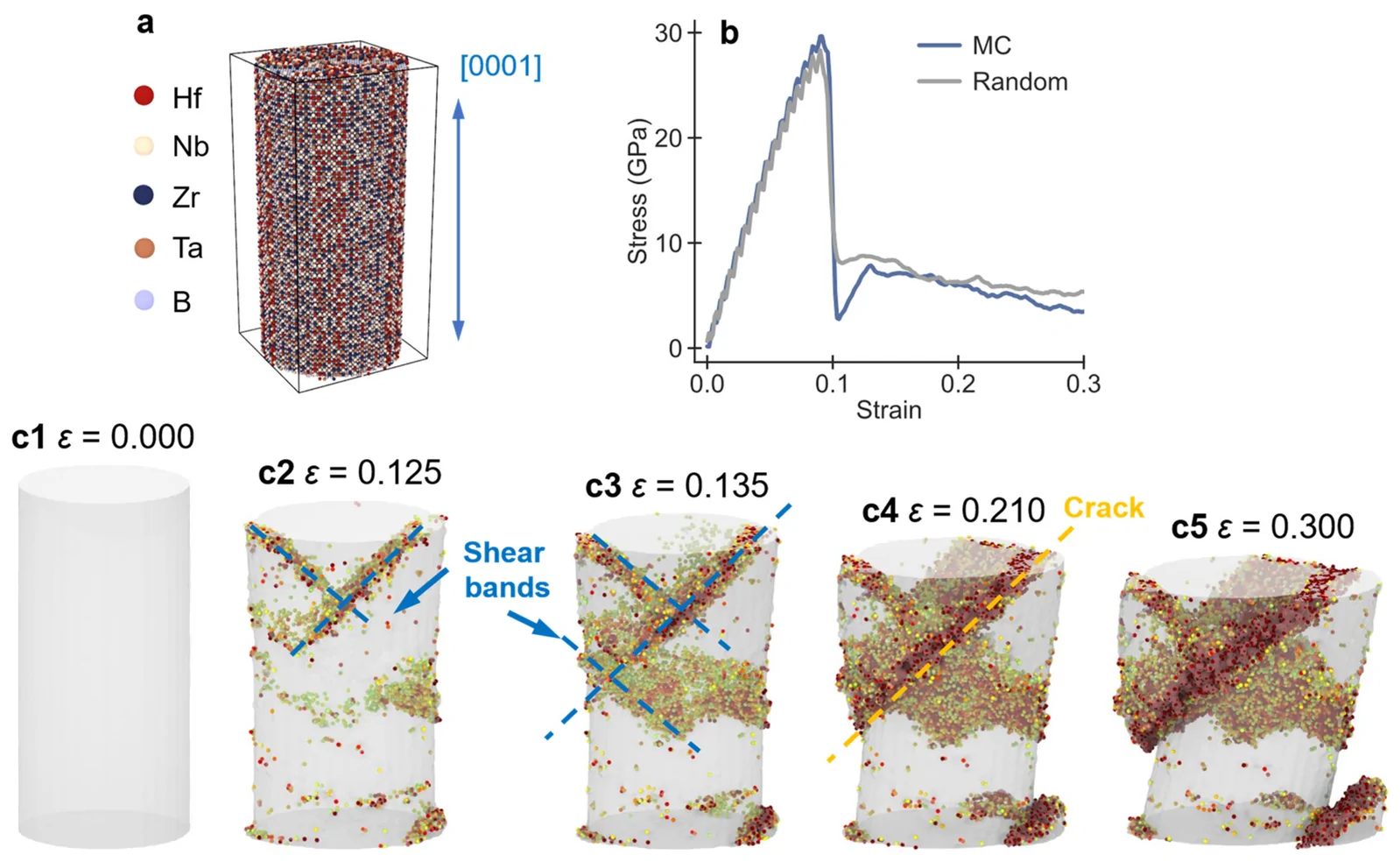
(**a**) A single crystalline HEB nanopillar under uniaxial compression. (**b**) The stress–strain responses of configurations with and without CSRO. (**c1**–**c5**) The nanostructural evolution during loading. Atoms are colored according to their atomic shear strain. Atoms with shear strain below 0.8 are excluded from the visualization.

The mechanical behavior of (HfNbTaZr)B_2_ is further assessed under uniaxial compression using the developed ABOP; the simulation model is displayed in Figure 10a. The temperature is maintained at 300 K during the whole loading process; a typical MD strain rate of 10^9^/s is used [46,52,53]. It should be noted that the strain rate of 10^9^/s adopted here, while typical for MD simulations, is higher than usual experimental loading conditions. However, this strain rate is low enough to ensure numerical convergence, as detailed in Section S1 of the Supplementary Material. To isolate the influence of CSRO, comparative simulations are conducted on both a random solid solution model and an MC-optimized model. As illustrated by the stress–strain responses in Figure 10b, both models display overall brittle failure behavior, consistent with prior assumptions [14,16,19,20,26]. The MC and Random models exhibit similar strength and flow stress, indicating a limited influence of CSRO on the mechanical response of the HEB. To understand this behavior, partial radial distribution functions (RDFs) of the two configurations are further compared, as shown in Figure S2 of the Supplementary Material. Although MC optimization noticeably redistributes the M-M pair correlations, consistent with the Warren–Cowley parameters in Figure 9b, the B-B and M-B pair correlations remain less influenced. The first B-B peak remains at ~1.83 Å, while the M-B first peaks remain at ~2.49–2.55 Å with only minor variations in peak intensity. These results indicate that cation CSRO mainly modifies chemical correlations within the metallic sublattice; the B-M nearest-neighbor coordination network exhibits no substantial reconstruction. This behavior is consistent with the previously proposed effect of the anion sublattice in high-entropy ceramics [1,54], which provides similar nearest-neighbor environments for different cations and accommodates differences in cation size and bond characters. In contrast, all constituent species in HEAs share an identical metallic lattice, and CSRO therefore directly redistributes their nearest-neighbor M-M environments. The HEA RDFs indeed exhibit much stronger changes after MC optimization (Figure S2d). This structural difference provides a plausible explanation for the much weaker influence of CSRO on the strength and deformation mechanism of the HEB.

In addition to the similar stress–strain responses, the nanostructural evolution processes of the Random and MC models are also analogous. Here, only the atomic structure of the Random model at varying deformation levels is shown in Figure 10(c1–c5). The yielding of the nanopillar is triggered via the formation of several local shear bands (Figure 10(c2)), which promptly release internal strain energy and cause a significant stress drop. These shear bands are oriented at ~45° to the pillar axis, corresponding to resolved maximum shear stress. During the post-yielding stage, deformation proceeds through multiplication and propagation of shear bands, as shown in Figure 10(c3). Shear strain localizes and accumulates within shear bands, which finally incubate cracks, as illustrated in Figure 10(c4,c5). Figure 10 displays a typical manner of brittle failure; the transformation from shear bands to cracks have been reported in many other brittle ceramics [14].

### 4.2. Nanoindentation

**Figure 11:**
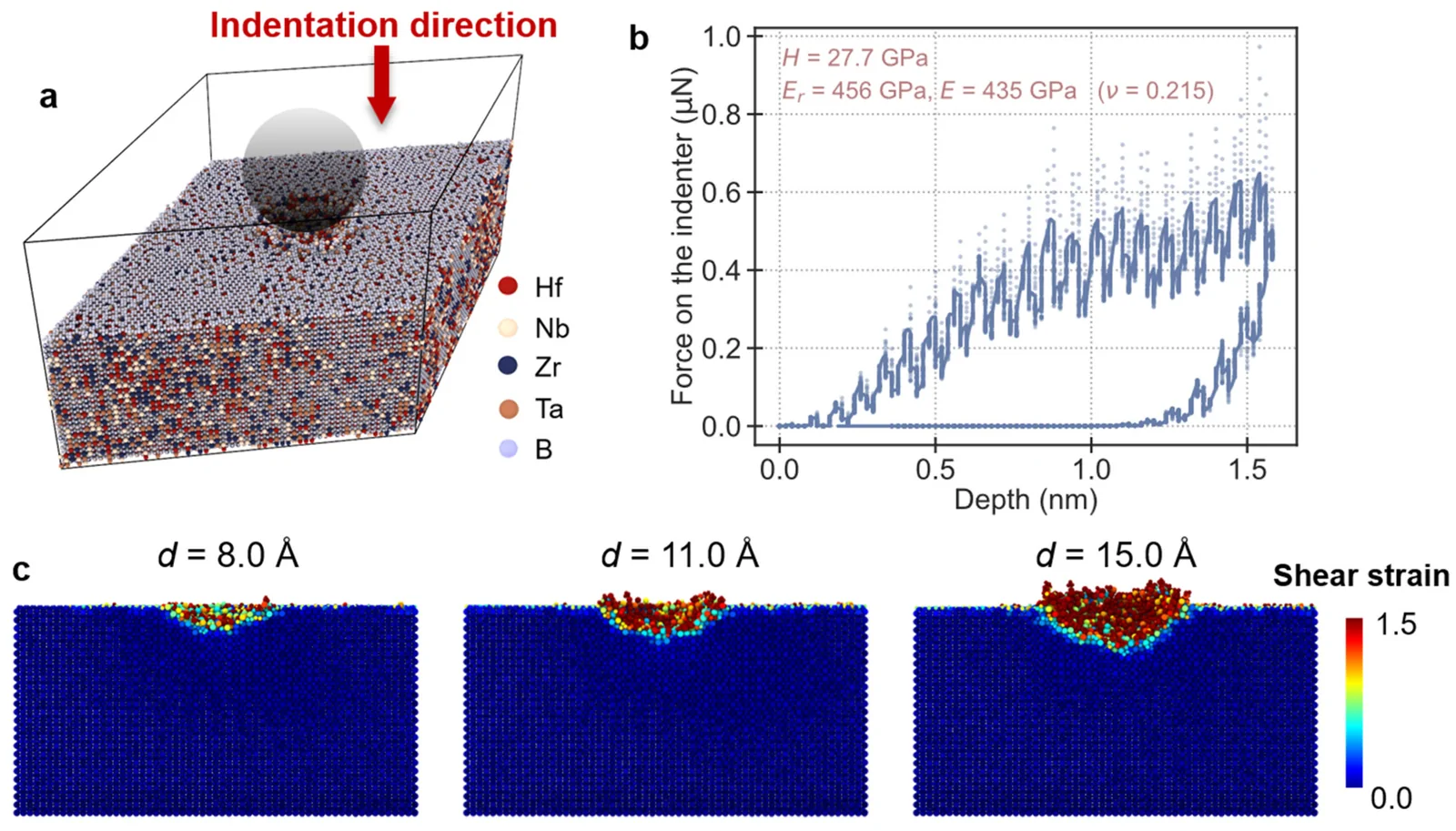
(**a**) The simulation model of nanoindentation using a spherical indenter. (**b**) The corresponding mechanical response during loading and unloading. (**c**) The atomic structural evolution during indentation. Atoms are colored according to local shear strain.

To facilitate a direct comparison with macroscopic mechanical properties of the HEB, we simulated a nanoindentation test on the (0001) surface (Figure 11a). A 60 × 60 × 25 supercell of single crystalline HEB is generated containing 270,000 atoms. The indentation is performed using a virtual spherical indenter with radius of 2.5 nm, which moves at a velocity of 20 m/s. The repulsive force between the indenter and nearby atoms can be expressed as *F*(*r*) = *Kθ*(*R* − *r*)(*R* − *r*)^2^, where *K* is a specified force constant which equals 3.3 eV/Å^3^ [14]. Considering the fact that the indenter radius and loading rates used in MD simulations differ from macroscale experimental settings, additional sensitivity tests are performed by varying the indentation velocity and the indenter radius (detailed in Section S3 of the Supplementary Material). Results indicate that both modulus and hardness are reasonably stable within the tested parameter range, which should be interpreted as an atomistic estimate of the intrinsic single-crystal response rather than direct reproductions of macroscopic measurements. Microstructural factors present in experimental polycrystalline HEBs, including porosity, GBs, texture, secondary phases, and other pre-existing defects, are not explicitly represented in this idealized single-crystal model and may contribute to the quantitative differences between atomistic and experimental measurements.

From the simulated force-displacement curve (Figure 11b), the Young’s modulus (*E*_ABOP_) and hardness (*H*_ABOP_) are extracted as 435 GPa and 27.7 GPa, respectively. In addition, *E*_ABOP_ and *H*_ABOP_ can be verified using theoretic methods. Based on the previously calculated elastic constants (Figure 9e), the mechanical properties can be evaluated analytically:

(4) $$B_{V}=\frac{2}{9}\left(C_{11}+C_{12}+2C_{13}+\frac{1}{2}C_{33}\right)$$

(5) $$B_{R}=\frac{\left(C_{11}+C_{12}\right)C_{33}-2C_{13}^{2}}{C_{11}+C_{12}+2C_{33}-4C_{13}}$$

(6) $$G_{v}=\frac{1}{15}\left(2C_{11}+C_{33}-C_{12}-2C_{13}\right)+\frac{1}{5}\left(2C_{44}+C_{66}\right)$$

(7) $$C^{2}=\left(C_{11}+C_{22}\right)C_{33}-2C_{13}^{2}$$

(8) $$G_{R}=\frac{5C^{2}C_{44}C_{66}}{2\left[3B_{V}C_{44}C_{66}+C^{2}\left(C_{44}+C_{66}\right)\right]}$$

(9) $$B_{H}=\frac{1}{2}\left(B_{V}+B_{R}\right)$$

(10) $$G_{H}=\frac{1}{2}\left(G_{V}+G_{R}\right)$$

(11) $$E=\frac{9B_{H}G_{H}}{3B_{H}+G_{H}}$$

(12) $$v=\frac{3B_{H}-2G_{H}}{2\left(3B_{H}+G_{H}\right)}$$

(13) $$H=2{\left(\frac{G_{H}^{3}}{B_{H}^{2}}\right)}^{0.585}-3$$

(14) $$K_{\mathrm{IC}}=V_{0}^{1/6}G_{H}{\left(\frac{B_{H}}{G_{H}}\right)}^{1/2},G_{\mathrm{IC}}=K_{\mathrm{IC}}^{2}\left(\frac{1\;-v^{2}}{E}\right).$$

The calculated values are listed in Table 3. The theoretical modulus *E* (481.18 GPa) and hardness *H* (26.30 GPa) are comparable to the values measured from the simulation. The Poisson’s ratio of 0.215 falls between typical values for strong covalent bonds (~0.1) and purely ionic materials (>0.25) [14,15], indicating a mixed bonding character in the HEB. Moreover, existing studies have reported the following ranges for the key mechanical properties [16,18,19,20,55]: bulk modulus *B* = 276–301 GPa, shear modulus *G* = 228–237 GPa, Young’s modulus *E* = 340–503 GPa, hardness *H* = 22–31.9 GPa, and fracture toughness *K*_IC_ = 3.72–4.37 MPa·m^1/2^. These consistent mechanical parameters confirm that the developed ABOP reliably captures the underlying physical correlations.

Additionally, Figure 11c illustrates the atomic-level structural evolution during indentation, in which atoms are colored according to local shear strain. Clearly, atoms with significant shear strain are located close to the indented surface region. No subsurface plastic zone is observed, indicating a markedly brittle nature. This phenomenon differs from that of certain hard ceramics exhibiting nanoscale plasticity; for instance, in the (HfNbZrTa)C HEC, a dislocation network penetrates the bulk material to a depth nearly twice the radius of the indenter tip [14].

### 4.3. Multiscale Cohesive Zone Modeling of Polycrystalline (HfNbTaZr)B_2_

**Figure 12:**
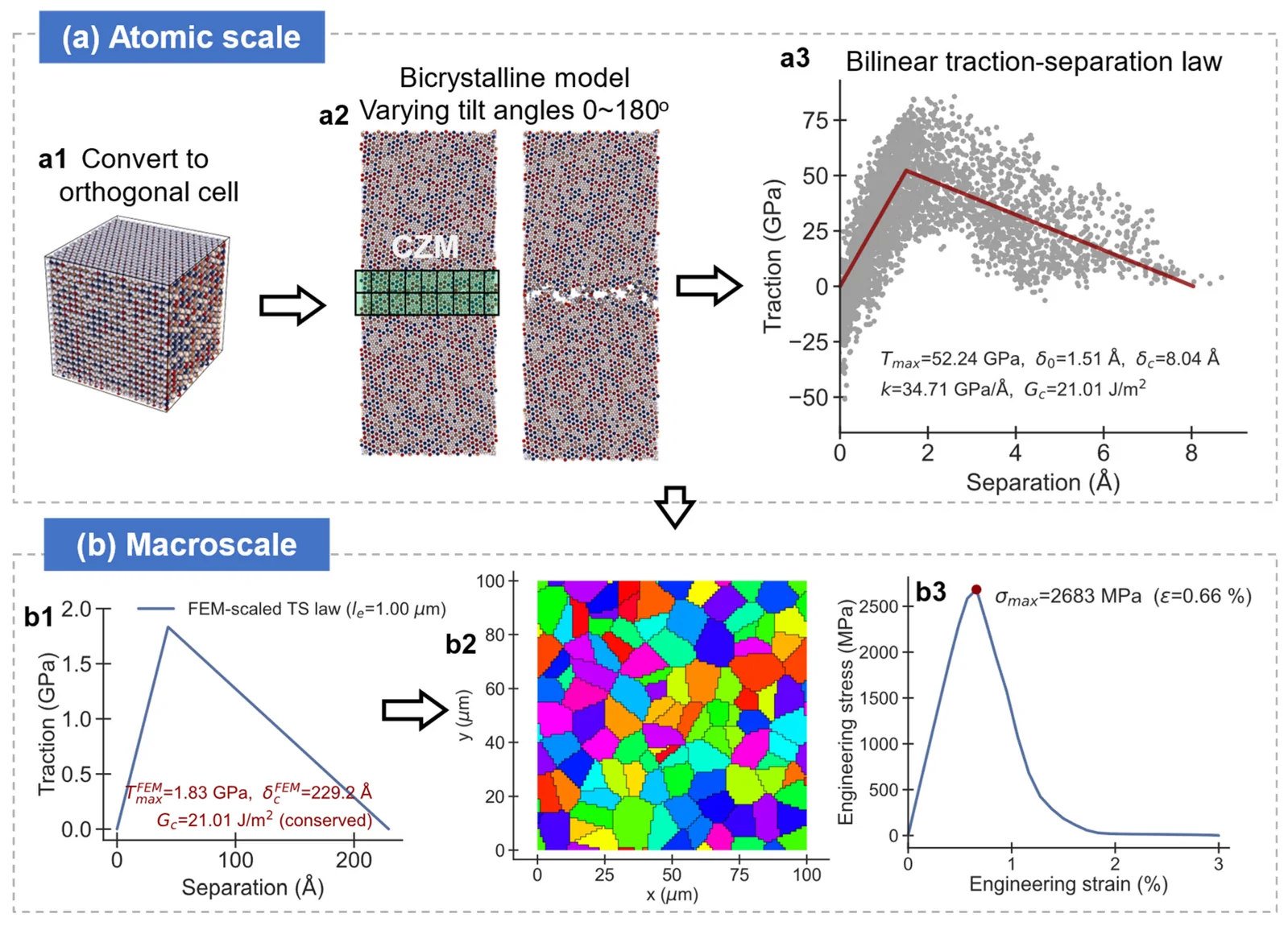
(**a1**–**a3**) Derivation of atomic-scale traction–separation (TS) law via bicrystalline fracture simulations. (**b1**) The corresponding macroscale TS law obtained based on the conservation of critical energy release rate. (**b2**) The FEM RVE polycrystalline model. (**b3**) The resulting FEM stress–strain curve.

The previous sections have demonstrated the reliability and applicability of the developed ABOP. Nonetheless, there exists a long-standing issue for MD simulation, namely, the MD results are difficult to directly compare with macroscale experimental tests or continuum modeling. Here, we propose a simple but effective multiscale method to connect MD to the finite element method (FEM); the workflow is shown in Figure 12. The hexagonal HEB supercell is converted to an orthogonal cell using ATOMSK (Figure 12(a1)), which searches for an equivalent orthogonal cell while preserving the periodicity of the configuration [56]. Numerous bicrystalline models are generated by combining two orthogonal cells at different tilt angles (Figure 12(a2)). The regions near the central GBs are divided into bins to record the evolution of displacement and force during subsequent uniaxial tensile deformation, as detailed in Refs. [57,58,59]. The traction–separation (TS) data from different bicrystalline models are gathered, as displayed in Figure 12(a3). An atomic-scale bilinear TS law is obtained by fitting these simulation data, characterized by the cohesive strength *T*_max_ = 52.24 GPa, the softening-onset separation *δ*_0_ = 1.51 Å, the critical separation *δ*_c_ = 8.04 Å, and the fracture energy *G*_c_ = 0.5(*T*_max_·*δ*_c_) = 21.01 J/m^2^. Notably, the critical energy release rate is close to the theoretical value in Table 3, indicating that it is reasonable to obtain the TS law from bicrystalline tensile simulations. It is also noteworthy that the resulting bilinear law should be interpreted as an effective representation of the sampled GB responses rather than the constitutive behavior of one specific boundary.

The atomic-scale TS law extracted from MD data quantifies the cohesive strength and fracture energy of GBs. However, directly applying this atomic-scale TS law to a macroscopic FEM calculation is impractical due to the large difference in scales between the atomistic process zone and standard FE meshes [59,60,61]. According to linear elastic fracture mechanics, the length of the process zone ahead of a crack tip *l*_cz_ is calculated as *l*_cz_ = *E*·*G*_c_/*T*_max_^2^. With a bulk elastic modulus *E* = 481 GPa (Table 3), *l*_cz_ is about 3.7 nm. Resolving this zone by at least *n* = 2–3 interface elements requires an element length *l*_e_ ≤ *l*_cz_/*n* ≈ 1.2 nm [59,60]; even for a two-dimensional 100 × 100 μm^2^ polycrystal, this would demand O(10^10^) elements, far beyond computational feasibility. Moreover, since the entire failure separation (*δ*_c_ ≈ 8 Å) is much smaller than a standard macroscopic load step, the elements will fail instantly within one step; the fracture energy is then released in an unstable burst.

To solve these problems, the TS law is rescaled while keeping the total fracture energy *G*_c_ constant. We select the element size *l*_e_ = 1 μm, which gives a rescaled strength $T_{\max}^{\prime }$ = 1.83 GPa and a larger separation $\delta_{c}^{\prime }$ = 22.9 nm. The rescaled process zone $l_{\mathrm{cz}}^{\prime }$ = 3.0 μm (Figure 12(b1)). This rescaling method is reasonable because brittle fracture is mainly controlled by the energy release rate *G*_c_ [62]. Similar multiscale approaches, which transfer the energy-based fracture mechanism from the atomic level to the engineering level, have previously been proven reliable [59,60,61]. Notably, a mesh-sensitivity analysis using element sizes of 0.5, 0.75, and 1.0 μm is conducted and shows that the peak stress, peak strain and post-peak response are only slightly influenced. The dissipated energy is also preserved within ~4% of the theoretical value calculated from the prescribed fracture energy *G*_c_, confirming that the adopted 1.0 μm discretization provides a sufficiently converged macroscopic response (Figure S3 of the Supplementary Material). Thereafter, a 100 × 100 μm^2^ representative volume element (RVE) containing 100 grains (mean grain size 11 μm, consistent with experiments [16,25,51]) is generated, as shown in Figure 12(b2). Periodic boundary conditions are applied to the RVE edges. The model is discretized with a 1 μm grid of bilinear plane-stress elements (CPS4R). To explicitly simulate intergranular fracture, zero-thickness cohesive elements (COH2D4) are inserted along all GBs. Consequently, the grains deform linearly and elastically; structural damage is governed by the cohesive interfaces. Crystallographic orientations are not explicitly assigned in the present continuum RVE. Instead, all grains are described using an isotropic linear elastic constitutive law, while the MD-derived responses of GBs are represented by an effective cohesive law. This idealization is adopted because the present model focuses on brittle intergranular fracture, which is consistent with the low fracture toughness and intergranular fracture behaviors reported for HEBs [63,64]. Consequently, the stochasticity of the RVE originates from the grain geometry and GB network. The effects of mesh size and grain distribution are further examined in Section S4 of the Supplementary Material.

**Figure 13:**
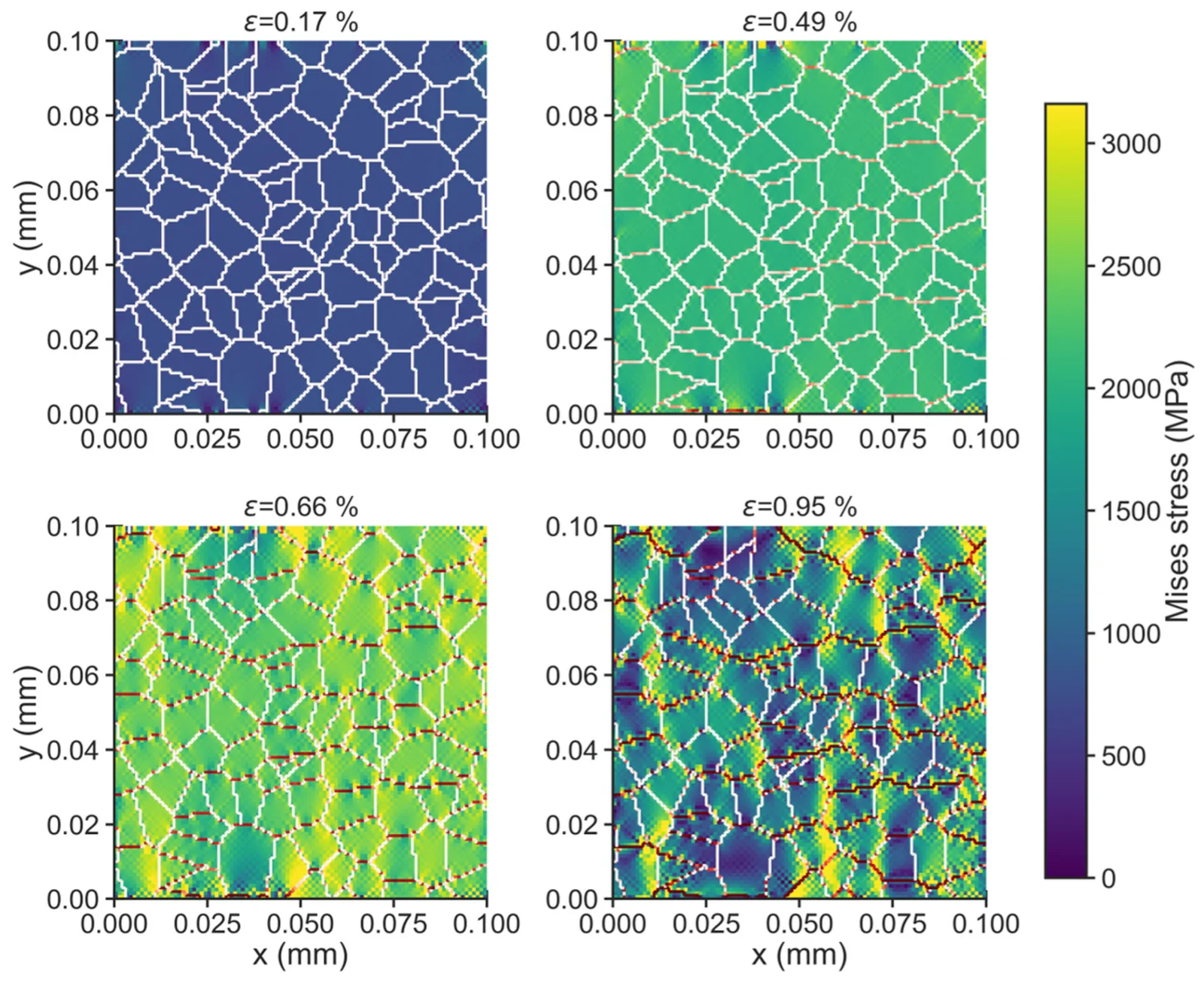
The Mises stress contour under different strain levels during tensile loading.

The engineering stress–strain curve (Figure 12(b3)) initially shows a linear elastic behavior, reaching a peak stress of 2683 MPa at a strain of 0.66%. This is followed by a sharp stress drop, where the load-carrying capacity falls below 20 MPa by 2% strain. This rapid failure indicates a brittle intergranular fracture, which is consistent with findings in the previous sections. Two aspects of this mechanical response are notable. First, the critical strain of 0.66% is slightly higher than the theoretical elastic limit of the grains (~0.55%), which is caused by the early softening of GBs that adds compliance before fracture. Second, the peak stress of 2683 MPa exceeds the assigned GB strength of 1.83 GPa. Because the load redistributes across the complex GB network during deformation, the overall strength differs from a simple average of individual GBs. The corresponding microstructural damage evolution is illustrated in Figure 13, including the Mises stress and GB damage across different deformation stages. During the initial loading stage (*ε* ≤ 0.49%), discrete damage begins at weaker GBs perpendicular to the tensile direction. As the stress approaches its peak (*ε* = 0.66%), these damaged GBs connect to form horizontal bands across several grains. The intact boundaries between these bands continue to carry the load, indicating that initial failure is driven by widespread microcracking rather than the propagation of a single dominant crack. Following the peak stress (*ε* ≥ 0.95%), the damage bands quickly merge into continuous cracks. As these cracks open along the GB network, the surrounding intact grains unload their stored elastic energy. The resulting fracture path is entirely intergranular, which is consistent with the fundamental assumption of the cohesive zone model. It should be noted that all cohesive interfaces in the continuum RVE employ the effective traction–separation law. Consequently, variations associated with individual GB crystallography, local chemistry, and segregation are not explicitly resolved. The present framework is intended to preserve the atomistically derived fracture energy and capture the collective intergranular failure response, while a boundary-resolved model with interface-specific cohesive laws would be required to explicitly describe such heterogeneity and will be studied in future work.

## 5. Conclusions

In this study, an analytical bond-order potential is developed for the complex Hf/Nb/Zr/Ta/B system to investigate the nanoscale structural and mechanical behaviors of HEBs. The training process against an extensive ab initio dataset is achieved via a DBGA parameterization. The main conclusions are listed as follows:

1. The developed ABOP successfully reproduces the structural, energetic, and mechanical properties of unary metals, binary and ternary intermetallics, and diborides over a wide range of strains. Additionally, a cross-validation test demonstrates that this potential ensures structural stability during finite-temperature dynamic simulations and captures the CSRO pattern.
2. Using the developed ABOP, simulations of single crystalline HEB nanopillars under uniaxial compression revealed a brittle failure behavior. Yielding and subsequent fracture are dominated by the formation, multiplication, and cracking of local shear bands oriented at approximately 45° to the loading axis. Unlike in HEAs, CSRO exerts a limited influence on the mechanical response of the HEB.
3. Simulated nanoindentation on the (0001) surface gives a Young’s modulus of 435 GPa and a hardness of 27.7 GPa, consistent with theoretical evaluations and existing experimental data. The structural evolution during indentation showed highly localized deformation without deep subsurface plastic zones, further denoting the intrinsic brittleness of the material.
4. A multiscale framework is established to bridge atomic-scale MD simulations with macroscopic FEM modeling. By extracting an atomic TS law from bicrystalline MD models and rescaling it with conserved fracture energy *G*_c_, the FEM-CZM simulations reproduce the brittle intergranular failure of polycrystalline HEB.

## Figures and Tables

**Table 1:** Configurations and related properties in training dataset.

Materials	Configurations	Properties
Metals	HCP, FCC and BCC polymorphs	Lattice constants, cohesive energies, equations of states, elastic constants and stress tensors
Intermetallics	Binary intermetallic compounds	Lattice constants, cohesive energies, equations of states and stress tensors
Unary diborides	HfB_2_, NbB_2_, TaB_2_ and ZrB_2_	Lattice constants, cohesive energies, equations of states, elastic constants and stress tensors
Binary diborides	HfNbB_4_, HfZrB_4_, HfTaB_4_, NbZrB_4_, NbTaB_4_, ZrTaB_4_	Lattice constants, cohesive energies, equations of states and stress tensors

**Table 2:** Comparison of ABOP-predicted vacancy formation and (0001) surface energy of (HfNbTaZr)B_2_ with ab initio reference values.

Property	ABOP (This Work)	Ab Initio
Hf vacancy formation energy (eV)	4.50 ± 0.29	4.69 ± 0.03
Nb vacancy formation energy (eV)	3.69 ± 1.06	4.06 ± 0.06
Zr vacancy formation energy (eV)	4.22 ± 0.78	3.94 ± 0.03
Ta vacancy formation energy (eV)	4.51 ± 1.29	4.78 ± 0.05
Average metal vacancy formation energy (eV)	4.23 ± 0.98	4.36 ± 0.93
B vacancy formation energy (eV)	0.86 ± 0.34	0.80 ± 0.18
Metal-terminated (0001) surface energy (J/m^2^)	1.84 ± 0.02	1.80
B-terminated (0001) surface energy (J/m^2^)	1.61 ± 0.04	1.97
(0001) cleavage work (J/m^2^)	3.45 ± 0.05	3.78

**Table 3:** The theoretically evaluated mechanical properties of the HEB.

*B*_V_ (GPa)	*B*_R_ (GPa)	*B*_H_ (GPa)	*G*_V_ (GPa)	*G*_R_ (GPa)	*G*_H_ (GPa)
281.21	280.93	281.97	199.95	196.19	198.07
*C*^2^ (GPa^2^)	*E* (GPa)	*v*	*H* (GPa)	*K*_IC_ (MPa·m^1/2^)	*G*_IC_ (J/m^2^)
3.1 × 10^5^	481.18	0.2147	26.30	3.38	22.70

## Data Availability

The original contributions presented in this study are included in the article/Supplementary Material. Further inquiries can be directed to the corresponding authors.

## Supplementary Materials

The following supporting information can be downloaded at: https://www.mdpi.com/article/10.3390/ma19194209/s1. Figure S1. The stress-strain curves of HEB nanopillar under uniaxial compression at strain rates of 5 × 10^11^/s and 10^9^/s, respectively. Configurations at 0.05, 0.10, 0.15 and 0.25 strains are selected and thermally relaxed for 100 ps with fixed strain levels; the resulting stress evolution is also displayed. Figure S2. Partial radial distribution functions (RDFs) of the Random and MC-optimized configurations. (a) B–B and (b) M–B pair correlations in the (HfNbZrTa)B_2_ HEB. (c) Representative M–M pair correlations in the HEB. (d) Representative M–M pair correlations in the HfNbZrTa HEA for comparison. The nearly unchanged B–B and M–B first-neighbor peaks indicate that cation CSRO produces only limited perturbation to the B-containing nearest-neighbor coordination structure of the HEB. Table S1. Sensitivity of the extracted Young’s modulus and hardness to indenter radius and indentation velocity. Figure S3. Mesh-size sensitivity of the macroscopic tensile response predicted by the CZM-FEM calculations. Engineering stress-strain curves obtained using varying element sizes are shown. Symbols indicate the peak stress of each curve. Figure S4. Mechanical responses of various polycrystalline RVEs of the HEB under uniaxial tensile loading. Table S2. Responses of five statistically equivalent RVE microstructural realizations. Figure S5. (a1) Simulation model for ideal shear. (a2) and (a3) represent the corresponding strain energy and shear stress as functions of shear strain, calculated using DFT and ABOP. (b1) and (b2) are the side view and the top view of a bicrystalline HEB model containing two ∑7(0001) twist GBs. (b3) The traction vs. distance curves during rigid separation, computed via DFT and ABOP, respectively. References [50,52,53,65,66,67,68,69,70,71] are cited in the Supplementary Materials.
