# Supplementary material for: Atomistic-to-Macroscopic Mechanical Modeling of (HfNbTaZr)B2 High-Entropy Ceramics via a Developed Interatomic Potential
Source: Materials (Basel). 2026 Oct 2;19(19):4209. doi: 10.3390/ma19194209 (PMC13666073; doi:10.3390/ma19194209)
Supplement: Supplementary file 1 [file materials-19-04209-s001.zip › materials-4561169-supplementary.pdf]

Supporting Information

Atomistic-to-Macroscopic Mechanical Modeling of (HfNbTaZr)B_2 High-Entropy Ceramics via a Developed Interatomic Potential

Yihan Wu [1,*], Yuan-Xin Dai [2], Xingjie Chen [3] and Pengfei Yu [1,*]

[1] School of Mechanical Engineering and Automation, Fuzhou University, Fuzhou 350108, China

[2] Key Laboratory of High-Performance Polymer Materials and Technology of MOE, Department of Polymer Science and Engineering, School of Chemistry and Chemical Engineering, Nanjing University, Nanjing 210023, China

[3] State Key Laboratory for Strength and Vibration of Mechanical Structures, School of Aerospace Engineering, Xi'an Jiaotong University, Xi'an, 710049, China

***Corresponding authors:**

wuyihan@fzu.edu.cn (Y Wu), yupengfei0422@fzu.edu.cn (P Yu)

S1. Loading strain rate

The compressive loading is performed at a strain rate of 10^9/s. Although it is common for MD, this high strain rate is apparently higher compared with typical experimental strain rates. Here the strain rate effect is analyzed from the following aspects:

1. Because of the small timestep used in MD simulations (typically from 1.0 fs to 10.0 fs [1-3]), a high strain rate is necessary to observe apparent deformation within the MD time scale. Meanwhile, extensive studies on strain rate effects have reported that strain rates lower than 5×10^9/s usually yield reliable results consistent with experimentally observed mechanical behaviors [4-8]. Additionally, a previous MD study clearly reported a critical strain rate of 2.5×10^{11}/s, below which atomic structure can fully relax between two successive displacement loading steps [4]. The strain rate 10^9/s is apparently lower than the above-mentioned critical strain rates.

2. We have performed additional tests on the strain rate sensitivity of the HEB. The nanopillar is uniaxially compressed under a 5×10^{11}/s strain rate; the resulting stress-strain curve is displayed in Fig. S1, which suggests superplasticity of the HEB at this strain rate. Specifically, the stress level continues to rise even at strain level higher than 20% and no crack is observed within the nanopillar. Such a phenomenon apparently conflicts with the covalent nature of the HEB. On the contrary, the HEB nanopillar under 10^9/s strain rate exhibits correct brittle behavior. Moreover, the configurations at 0.05, 0.10, 0.15 and 0.25 strains are selected and thermally relaxed for 100 ps. The strain level is held fixed during relaxation and the variation of stress is recorded [9, 10]. Results in Fig. S1 reveal that the stress level drops to approach that under the lower strain rate, indicating a strain rate of 10^9/s is enough to reach convergence.

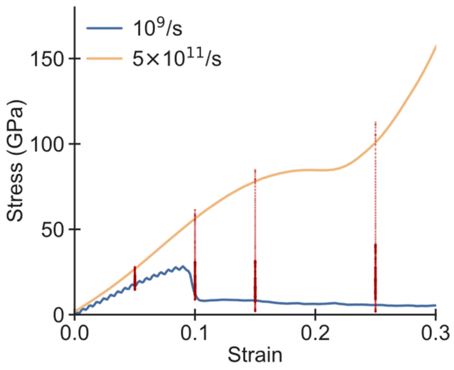

Fig. S1 The stress-strain curves of HEB nanopillar under uniaxial compression at strain rates of 5×10^{11}/s and 10^9/s, respectively. Configurations at 0.05, 0.10, 0.15 and 0.25 strains are selected and thermally relaxed for 100 ps with fixed strain levels; the resulting stress evolution is also displayed.

S2. Partial radial distribution function analysis of CSRO

To further examine the influence of cation CSRO in HEB, partial radial distribution functions (RDFs) are calculated for the Random and MC-optimized HEB configurations. As shown in Figs. S2a and S2b, the B–B and M–B RDFs of the two models almost overlap within the first coordination shell. The first B–B peak remains at approximately 1.83 Å, while the first M–B peaks remain at approximately 2.49–2.55 Å, with only minor variations in peak intensity. In contrast, appreciable redistributions are observed for M–M partial RDFs (Fig. S2c), consistent with the cation CSRO identified from the Warren–Cowley parameters (Fig. 9 of the main text). These results indicate that MC optimization primarily changes chemical correlations within the cation sublattice while preserving the B-containing nearest-neighbor coordination structure.

For comparison, the partial M–M RDFs of the HfNbZrTa HEA are shown in Fig. S2d. Much stronger redistribution of the first-neighbor M–M environments occurs after MC optimization, because all constituent species share an identical metallic lattice. This comparison highlights the unique structural influence of CSRO in the HEB and HEA.

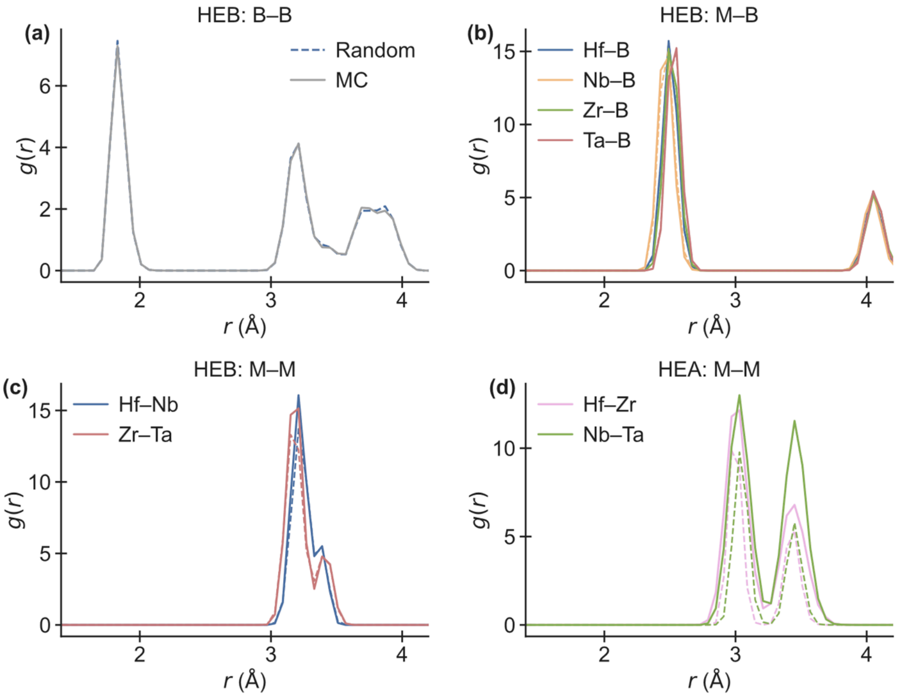

Fig. S2 Partial radial distribution functions (RDFs) of the Random and MC-optimized configurations. (a) B–B and (b) M–B pair correlations in the $(HfNbZrTa)B_2$ HEB. (c) Representative M–M pair correlations in the HEB. (d) Representative M–M pair correlations in the HfNbZrTa HEA for comparison. The nearly unchanged B–B and M–B first-neighbor peaks indicate that cation CSRO produces only limited perturbation to the B-containing nearest-neighbor coordination structure of the HEB.

S3. Sensitivity of nanoindentation results to indenter radius and loading velocity

Table S1 Sensitivity of the extracted Young's modulus and hardness to indenter radius and indentation velocity.

R **(nm)**	***v*** **(m/s)**	***E*** **(GPa)**	**Δ*E*** **(%)**	***H*** **(GPa)**	**Δ*H*** **(%)**
2.5	5	454.8	+4.5	25.0	-9.7
2.5	10	450.5	+3.6	25.6	-7.6
2.5	20	435.0	0	27.7	0
2.5	40	404.0	-7.1	29.3	+5.8
5.0	20	429.4	-1.3	25.6	-7.6
5.0	40	403.9	-7.1	28.6	+3.2

S4. Numerical robustness of the CZM-FEM modeling

S4.1 Mesh-size sensitivity

As shown in Fig. S3, all three meshes exhibit essentially the same macroscopic response. The peak strain remains 0.66%, while the peak stresses are 2,683, 2,754, and 2,852 MPa for l_e = 1.0, 0.75, and 0.5 μm, corresponding to a maximum variation of 6.3%. The three simulations also exhibit the same rapid post-peak stress reduction and nearly complete loss of load-carrying capacity. The strain at which the stress decreases below 10% of the peak value varies from 1.50% to 1.39%.

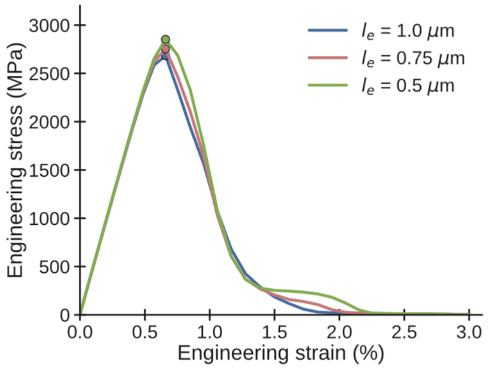

Fig. S3 Mesh-size sensitivity of the macroscopic tensile response predicted by the CZM-FEM calculations. Engineering stress-strain curves obtained using varying element sizes are shown. Symbols indicate the peak stress of each curve.

S4.2 Sensitivity to RVE microstructural realization

In the present FEM model, crystallographic grain orientations are not explicitly represented. All grains are described using an isotropic linear-elastic constitutive law. The traction-separation responses obtained from bicrystalline MD simulations are represented by an effective cohesive law. Consequently, the stochasticity of the RVE originates from the polycrystalline geometry, including grain morphology, GB connectivity, and GB inclination relative to the loading direction.

To examine the statistical representativeness of the selected RVE, four additional

microstructural realizations are generated using independently randomized grain nucleation positions. All five RVEs have the same dimensions (namely, 100×100 μm^2), number of grains (i.e., 100 grains), target grain-size distribution, material properties, cohesive parameters, mesh size, periodic boundary conditions, and loading history.

As shown in Fig. S4, the five RVEs exhibit closely comparable macroscopic stress-strain responses. The average peak stress is 2,721 ± 27 MPa, and the peak strain is 0.66% for every RVE. The strain at which the stress decreases below 10% of its peak value is 1.39 ± 0.08 % (Table S2). Although the detailed crack paths differ because of variations in GB topology, all RVE configurations exhibit the same characteristic sequence of GB damage, coalescence of damaged boundaries, and final intergranular brittle fracture. These results indicate that the macroscopic response and failure mechanism are representative of the present class of RVE microstructures.

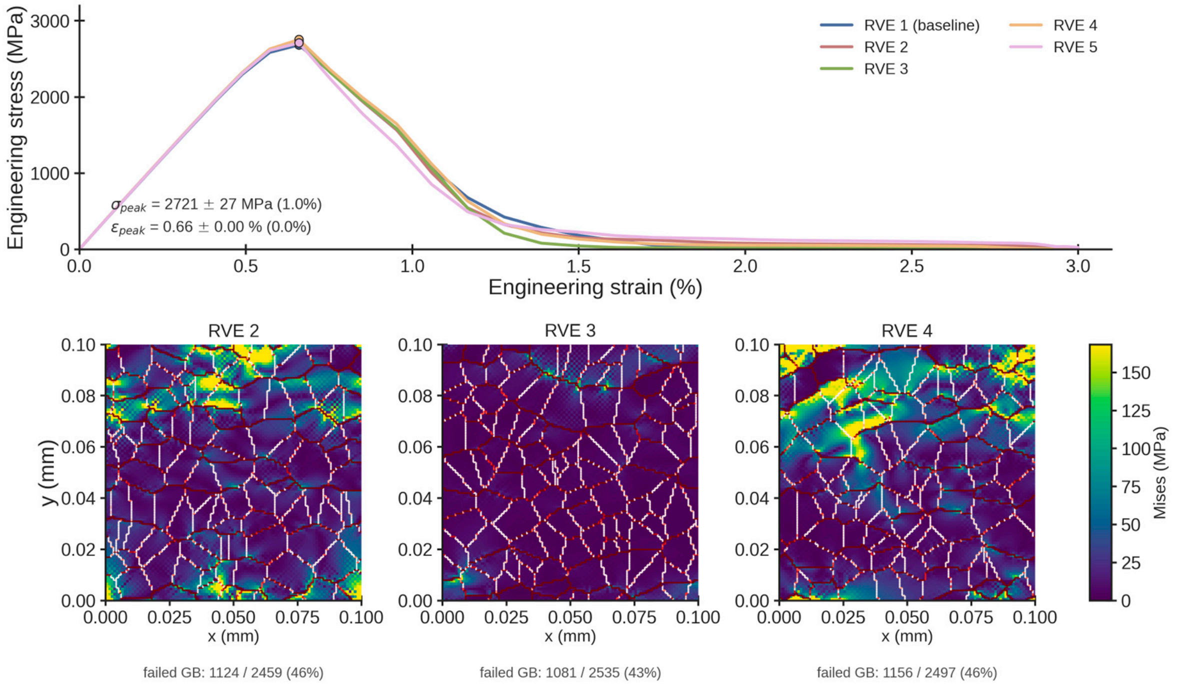

Fig. S4 Mechanical responses of various polycrystalline RVEs of the HEB under uniaxial tensile loading.

Table S2 Responses of five statistically equivalent RVE microstructural realizations.

Configuration	Peak stress (MPa)	Peak strain (%)	Strain at σ < 10% σ_{peak} (%)
RVE1 (main text)	2,683	0.66	1.50
RVE2	2,735	0.66	1.39
RVE3	2,725	0.66	1.28
RVE4	2,754	0.66	1.39
RVE5	2,710	0.66	1.39
**Mean ± STD**	**2,721 ± 27**	**0.66 ± 0.00**	**1.39 ± 0.08**

S5. Independent validation under ideal shear and GB separation

S5.1 Ideal shear deformation

A compact equiatomic (HfNbZrTa)B_2 configuration is employed to assess the nonlinear shear response (Fig. S5a1). The same initial structure and shear deformation path are used in the DFT and ABOP calculations. At each shear strain level, structural relaxation is performed using energy minimization, after which the corresponding strain energy and shear stress are extracted. This shear test therefore provides a direct comparison of the nonlinear mechanical response predicted by DFT and ABOP.

As shown in Fig. S5, the two stress-strain curves remain closely consistent over the investigated strain range. The maximum shear stresses are 57.9 GPa for DFT and 57.1 GPa for ABOP. Both curves exhibit shear instability at approximately 0.25-0.30 strain.

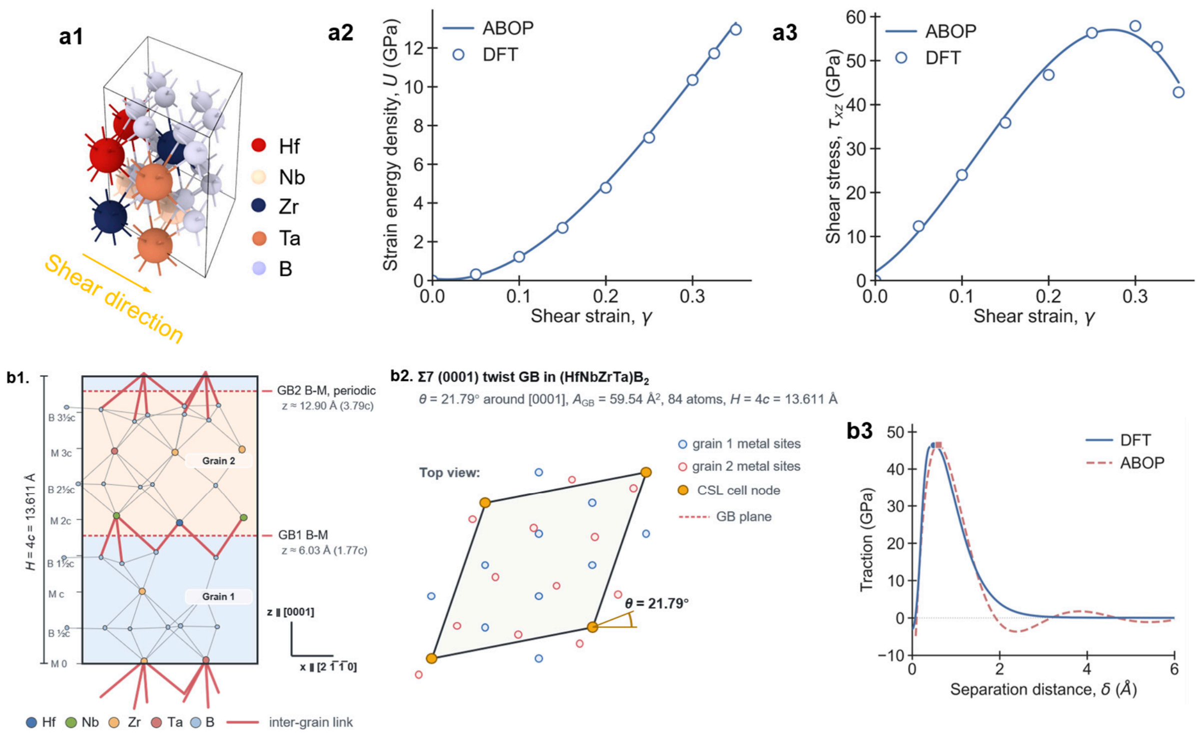

Fig. S5 (a1) Simulation model for ideal shear. (a2) and (a3) represent the corresponding strain energy and shear stress as functions of shear strain, calculated using DFT and ABOP. (b1) and (b2) are the side view and the top view of a bicrystalline HEB model containing two ∑7(0001) twist GBs. (b3) The traction vs. distance curves during rigid separation, computed via DFT and ABOP, respectively.

S5.2 Representative GB separation

A compact representative HEB bicrystal is constructed to enable direct DFT validation at an affordable computational cost. Because direct DFT calculations of the bicrystalline models containing tilt GBs (as shown in Fig. 12 of the main text) would be computationally prohibitive, a compact HEB bicrystal containing twist GBs is constructed specifically for this independent validation (Figs. S5b1 and S5b2). The initial GB configuration is first relaxed, after which rigid separation is imposed without further structural relaxation. Identical atomic configurations at each separation distance are evaluated using DFT and ABOP. The resulting interfacial forces are normalized by the GB area to obtain the traction.

The DFT and ABOP traction-separation responses exhibit closely comparable behavior. The maximum cohesive tractions are 46.40 and 46.51 GPa, respectively, corresponding to a difference

of only ~ 0.25%. The ABOP peak occurs at a slightly larger separation (~0.60 Å) than the DFT value (~0.49 Å). After the primary decohesion stage, some quantitative differences occur in the low-traction tail, but both calculations consistently indicate a rapid loss of GB load-carrying capacity.

References:

[1] **P.F. Batcho, D.A. Case, T. Schlick**, *Optimized particle-mesh Ewald/multiple-time step integration for molecular dynamics simulations.* The Journal of Chemical Physics, 2001. **115**(9): p. 4003-4018.

[2] **J.C. Sweet, R.J. Nowling, T. Cickovski, C.R. Sweet, V.S. Pande, J.A. Izaguirre**, *Long Timestep Molecular Dynamics on the Graphical Processing Unit.* Journal of Chemical Theory and Computation, 2013. **9**(8): p. 3267-3281.

[3] **X. Li, W. Yang**, *Multiple time step molecular dynamics simulation for interaction between dislocations and grain boundaries.* Acta Mechanica Sinica, 2005. **21**(4): p. 371-379.

[4] **S.C. Chowdhury, B.Z. Haque, J.W. Gillespie**, *Molecular dynamics simulations of the structure and mechanical properties of silica glass using ReaxFF.* Journal of Materials Science, 2016. **51**(22): p. 10139-10159.

[5] **F.G. Sen, A.T. Alpas, A.C.T. van Duin, Y. Qi**, *Oxidation-assisted ductility of aluminium nanowires.* Nature communications, 2014. **5**: p. 3959.

[6] **S. Ding, X. Wang**, *Strain rate and temperature effects on the mechanical properties of TiN/VN composite: Molecular dynamics study.* Journal of Alloys and Compounds, 2020. **814**: p. 152151.

[7] **L. Chang, C.-Y. Zhou, H.-X. Liu, J. Li, X.-H. He**, *Orientation and strain rate dependent tensile behavior of single crystal titanium nanowires by molecular dynamics simulations.* Journal of Materials Science & Technology, 2018. **34**(5): p. 864-877.

[8] **Y.-H. Wen, Z.-Z. Zhu, R.-Z. Zhu**, *Molecular dynamics study of the mechanical behavior of nickel nanowire: Strain rate effects.* Computational Materials Science, 2008. **41**(4): p. 553-560.

[9] **Y. Wu, Z. Bai, G. Yan, W. Yu, S. Shen**, *Size-dependent mechanical responses of twinned Nanocrystalline HfNbZrTi refractory high-entropy alloy.* International Journal of Refractory Metals and Hard Materials, 2024. **125**: p. 106885.

[10] **J. Li, Y. Wu, Z. Bai, W. Yu, S. Shen**, *Nanostructure-property relation of Σ5 grain boundary in HfNbZrTi high-entropy alloy under shear.* Journal of Materials Science, 2023. **58**(15): p. 6757-6774.
